# Virtual Screening–Guided Identification of Candidate USP7-Inhibitory Scaffolds Exhibiting Antiproliferative Activity

**DOI:** 10.3390/ph19081219

**Published:** 2026-08-03

**Authors:** Rita I. Oliveira, Caio Franco, Miguel Mano, Ana S. Leal, Jorge A. R. Salvador

**Affiliations:** 1 Laboratory of Pharmaceutical Chemistry, Faculty of Pharmacy, University of Coimbra, 3000-548 Coimbra, Portugal; rita.oliveira@cnc.uc.pt; 2 Center for Neuroscience and Cell Biology, Center for Innovative Biomedicine and Biotechnology, University of Coimbra, 3060-197 Cantanhede, Portugal; caiohaddadfranco@gmail.com (C.F.); mano@ci.uc.pt (M.M.); 3 Division of Hematology and Oncology, Indiana University School of Medicine, Indianapolis, IN 46202, USA; anmendes@iu.edu; 4 Indiana University Melvin and Bren Simon Comprehensive Cancer Center, Indiana University, Indianapolis, IN 46202, USA

**Keywords:** virtual screening, pharmacophore modelling, molecular docking, drug discovery, USP7 inhibitors, antitumor

## Abstract

**Background/Objectives**: Ubiquitin-specific protease 7 (USP7) is a deubiquitinating enzyme that regulates multiple oncogenic and tumor-suppressive pathways and has emerged as a promising target for anticancer drug discovery. However, most reported USP7 inhibitors belong to a limited number of structural classes, and no USP7-targeted therapy has yet reached clinical application. This study aimed to identify structurally diverse candidate USP7-inhibitory scaffolds with antiproliferative activity using an integrated computational and experimental screening strategy. **Methods**: A drug discovery workflow combining pharmacophore modelling, structure-based virtual screening, molecular docking, and in silico pharmacokinetic assessment was employed to identify candidate USP7 inhibitors. Selected compounds were evaluated for inhibition of recombinant USP7 and for antiproliferative activity in a panel of human cancer cell lines. Molecular docking analyses were performed to investigate predicted binding modes. **Results**: The primary screening campaign identified four active compounds (>50% inhibition at 10 µM) and twenty-one weakly active compounds (10–49% inhibition at 10 µM), including structurally distinct approved drugs and compounds from an in-house chemical library. The four active compounds were subsequently validated by dose–response assays against recombinant USP7 and exhibited micromolar inhibitory activity. These candidate USP7-inhibitory scaffolds also exhibited antiproliferative activity across cancer cell lines with different molecular backgrounds. Docking studies predicted binding within a pharmacologically relevant region of the USP7 catalytic cleft and revealed putative interactions with key residues involved in ligand recognition. Among the compounds evaluated, rafoxanide demonstrated the most favorable combination of predicted USP7 binding and antiproliferative activity. **Conclusions**: This integrated virtual screening and experimental validation approach enabled the identification of candidate USP7-inhibitory scaffolds with preliminary antiproliferative activity. The identified hits include approved drugs with previously unreported USP7 inhibitory activity, as well as underexplored scaffolds that expand the chemical space of candidate USP7-targeting molecules. These candidate scaffolds warrant further medicinal chemistry optimization, orthogonal validation, and mechanistic characterization to establish their potential as USP7-targeted anticancer agents.

## 1. Introduction

The ubiquitin–proteasome system (UPS) is a tightly regulated nonlysosomal intracellular protein degradation pathway that maintains cellular proteostasis through the coordinated ubiquitination and degradation of intracellular protein [1,2]. Beyond its canonical role in protein turnover, the UPS regulates numerous cellular processes, including cell-cycle progression [3], DNA replication and repair [4], transcriptional regulation [5], apoptosis [6], immune signaling [7], and epigenetic modulation [8]. Protein ubiquitination is a dynamic and reversible post-translational modification mediated through a sequential enzymatic cascade involving ubiquitin-activating (E1), ubiquitin-conjugating (E2), and ubiquitin ligase (E3) enzymes, which collectively determine substrate specificity and ubiquitin chain assembly [9]. Counterbalancing this process are deubiquitinating enzymes (DUBs), a diverse family of proteases that remove ubiquitin moieties from substrate proteins, thereby regulating their stability, localization, and functional activity while maintaining ubiquitin homeostasis within the pool of cellular ubiquitin [10]. Among the approximately 100 human DUBs identified to date, ubiquitin-specific protease 7 (USP7), also known as herpesvirus-associated ubiquitin specific protease (HAUSP), has emerged as one of the most extensively characterized and therapeutically relevant members of the USP family [11,12,13].

USP7 is a multidomain cysteine protease composed of an N-terminal TRAF domain, a central catalytic domain, and C-terminal ubiquitin-like domains that collectively regulate substrate recognition and catalytic activity [14,15,16]. Through the deubiquitination of key oncogenic and tumor suppressor proteins (e.g., MDM2 [17], p53 [17], PTEN [18]), transcriptional factors (e.g., FOXP3 [19]), and several epigenetic regulators (e.g., DNMT1 [20], Tip60 [21]), USP7 plays a pivotal role in tumor-associated signaling networks. Aberrant USP7 expression has been reported in multiple malignancies, including breast cancer [22,23], lung cancer [24], colorectal cancer [25], prostate cancer [26], acute myeloid leukemia [27] and multiple myeloma [28], where elevated USP7 levels are frequently associated with tumor progression and poor clinical prognosis. Consequently, pharmacological inhibition of USP7 has attracted considerable interest as a promising anticancer strategy capable of restoring tumor suppressor activity and disrupting oncogenic signaling pathways.

Over the past decade, substantial efforts have been directed toward the development of selective small-molecule USP7 inhibitors. Early compounds such as HBX41108 [29], P22077 [30], P5091 [28], and related analogues [31,32] demonstrated limited selectivity and modest potency, largely due to the absence of detailed structural information regarding inhibitor binding. The elucidation of co-crystal structures of USP7 bound to inhibitors including FT671 (PDB ID: 5NGE) [33], FT827 (PDB ID: 5NGF) [33], GNE-6640 (PDB ID: 5UQV) [34], and GNE-6776 (PDB ID: 5UQX) [34] represented a major advance in the field, enabling structure-guided optimization and facilitating the development of highly potent inhibitors with nanomolar activity. Despite these advances, most reported USP7 inhibitors remain structurally related. To date, no USP7 inhibitor has entered clinical development, likely reflecting the difficulty of balancing potency, selectivity, and drug-like properties within existing chemotypes [35]. Consequently, identifying novel and chemically diverse USP7 inhibitor scaffolds remains an important goal in developing therapeutically viable USP7-targeted agents.

In this context, structure-based virtual screening has emerged as a powerful strategy for efficient exploration of the chemical space and the identification of structurally diverse bioactive scaffolds in early-stage drug discovery [36,37]. Therefore, in the present study, we implemented an integrated computational and experimental workflow combining pharmacophore modelling, structure-based virtual screening, molecular docking, in silico pharmacokinetic assessment, and in vitro biological evaluation. This coordinated strategy enabled the efficient prioritization and experimental validation of candidate USP7-inhibitory scaffolds exhibiting preliminary antiproliferative activity.

## 2. Results

### 2.1. Potential Druggable Hot Spots of USP7

To investigate the druggability of the USP7 catalytic cleft and identify regions likely to contribute to ligand recognition, four inhibitor-bound crystal structures (PDB ID: 5N9T, 6M1K, 6VN2, and 6VN6) were selected for analysis. Ligand–receptor interactions observed in the crystallographic complexes are summarized in Table S1 (Supporting Information). Hydrogen-bond interactions were predominantly established with Asp295, Val296, Gln297, Gln351, Leu406, Arg408, Phe409, and Tyr465, whereas aromatic contacts involved Gln351, Met407, Phe409, His456, and Tyr514. Ionic interactions were primarily mediated by Asp295.

To further characterize the binding pocket, the selected crystal structures were analyzed using the FTMap computational mapping server. Multiple consensus sites (CSs) were identified within the substrate-binding pocket of each structure. Comparison of the predicted hot spots with the binding modes of the co-crystallized inhibitors revealed a strong spatial overlap between the highest-ranking CSs and the ligand-occupied regions of the catalytic cleft (Table S2 and Figure S1, Supporting Information). Although several secondary hot spots were also detected, a subset of weak and spatially isolated consensus sites showed no overlap with the crystallographic ligands and were therefore excluded from further analysis.

The overall distribution of probe clusters showed remarkable agreement with the experimentally observed ligand-binding modes, supporting the robustness of the FTMap analysis. Superposition of the main and secondary hot spots across the four crystal structures (Figure S2, Supporting Information) revealed a conserved pattern of favorable interaction regions within the USP7 binding pocket.

**Figure 1:**
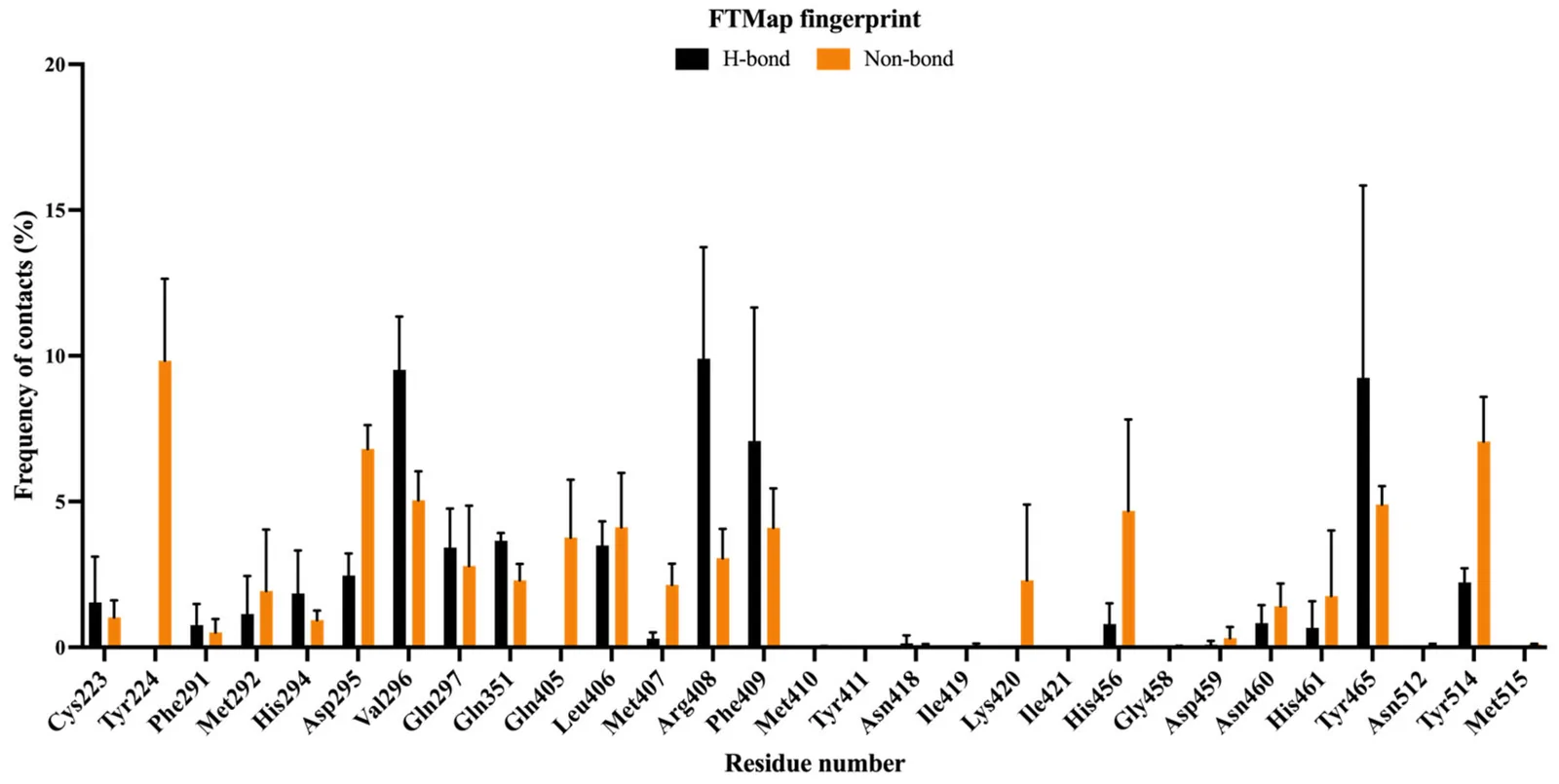
Interaction fingerprint map depicting the percentage contributions of hydrogen-bond and non-bonded contacts between small-molecule probes and residues within the USP7 substrate-binding cleft (PDB IDs: 5N9T, 6M1K, 6VN2, and 6VN6), as determined by FTMap analysis. Error bars represent the standard deviation of the percentage values across the different crystal structures. Black and orange bars correspond to hydrogen-bond and non-bonded interactions, respectively.

FTMap additionally enabled the characterization of residue–probe contacts and their relative frequencies. Figure 1 presents the resulting mapping fingerprint, summarizing the contribution of individual residues to non-bonded and hydrogen-bond interactions across all four crystal structures (PDB ID: 5N9T, 6M1K, 6VN2, and 6VN6). Residues exhibiting the highest frequencies of non-bonded contacts corresponded to the most populated probe clusters and were therefore considered key contributors to ligand recognition. The residues most strongly associated with probe binding included Tyr224, Asp295, Val296, Gln297, Gln351, Leu406, Arg408, Phe409, His456, Tyr465, and Tyr514. Notably, these residues are also prominently represented in the interaction fingerprints of the co-crystallized inhibitors (Table S1, Supporting Information), further supporting their importance in molecular recognition.

**Figure 2:**
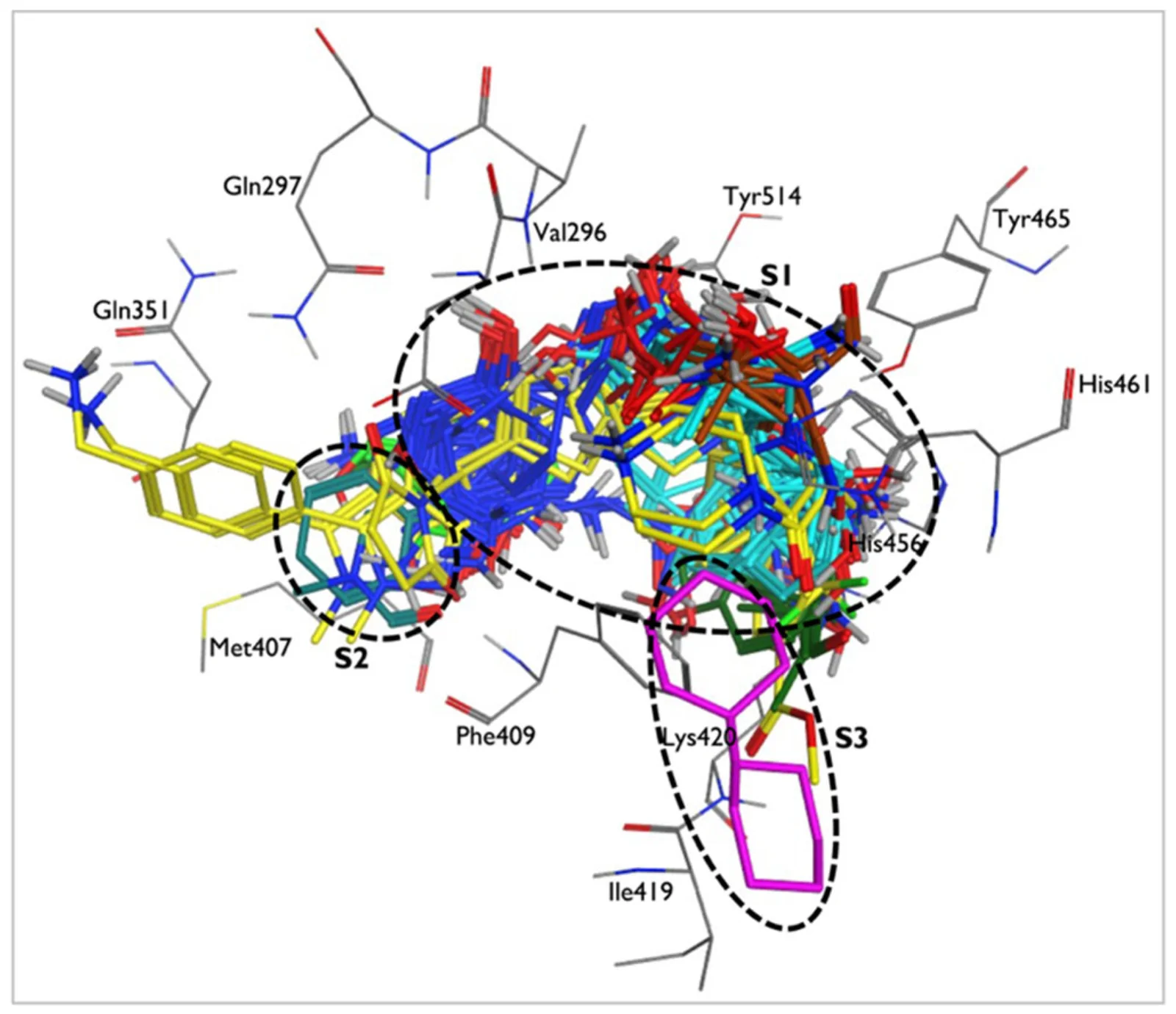
Identification of subpockets Sp1–Sp3 within the USP7 substrate-binding site. Residues forming the binding site are depicted as grey sticks. Probe clusters at consensus sites are shown as sticks in multiple colors and are superimposed with the four co-crystallized ligands, represented as yellow sticks.

Analysis of the spatial distribution of the CSs allowed to organize the substrate-binding pocket of USP7 into three subpockets (Figure 2). Subpocket 1 (Sp1) comprises the principal hot-spot region and contains the highest density of consensus sites, including the main hot spots identified in all four crystal structures. This region is surrounded by Tyr224, Asp289, Met292, Gln293, Asp295, Val296, Gln297, Arg408, Phe409, His456, His461, Tyr465, and Tyr514, and exhibits extensive overlap with all co-crystallized ligands. Subpocket 2 (Sp2), located near the entrance of the binding pocket, is defined by secondary hot spots positioned in proximity to Gln351 and Met407. Subpocket 3 (Sp3) consists of a smaller auxiliary region located near Gln417, Asn418, Ile419, Lys420, Ile421, Asn422, Asp423, and His501.

Collectively, these results identify a conserved network of hot spot residues that appears to govern ligand recognition within the USP7 catalytic cleft. The strong agreement between FTMap-derived interaction fingerprints and experimentally observed ligand-binding modes highlights the relevance of these residues as key determinants of molecular recognition. Furthermore, the identification of a dominant ligand-recognition region (Sp1) together with adjacent auxiliary subpockets provides a structural framework for the interpretation of inhibitor binding. Therefore, FTMap analysis was used as an orthogonal approach to validate and support the selection of key interaction features incorporated into the subsequent structure-based pharmacophore models.

### 2.2. Pharmacophore Modelling and Validation

The pharmacophore hypotheses created comprise a total of six and five key pharmacophoric features for models A and B, respectively, distributed along the C-terminal tail binding site of ubiquitin-aldehyde (Ubal) within the USP7 substrate-binding pocket. Excluded volumes were generated to reflect the steric constraints of the binding pocket, based on active-site residues located within 4.5 Å radius of the ligand atoms. The inclusion of excluded volumes enables the elimination of compounds that would sterically clash with USP7 residues. Subsequently, the tolerance radius of each pharmacophoric feature was adjusted to achieve an optimal balance between sensitivity and specificity.

**Figure 3:**
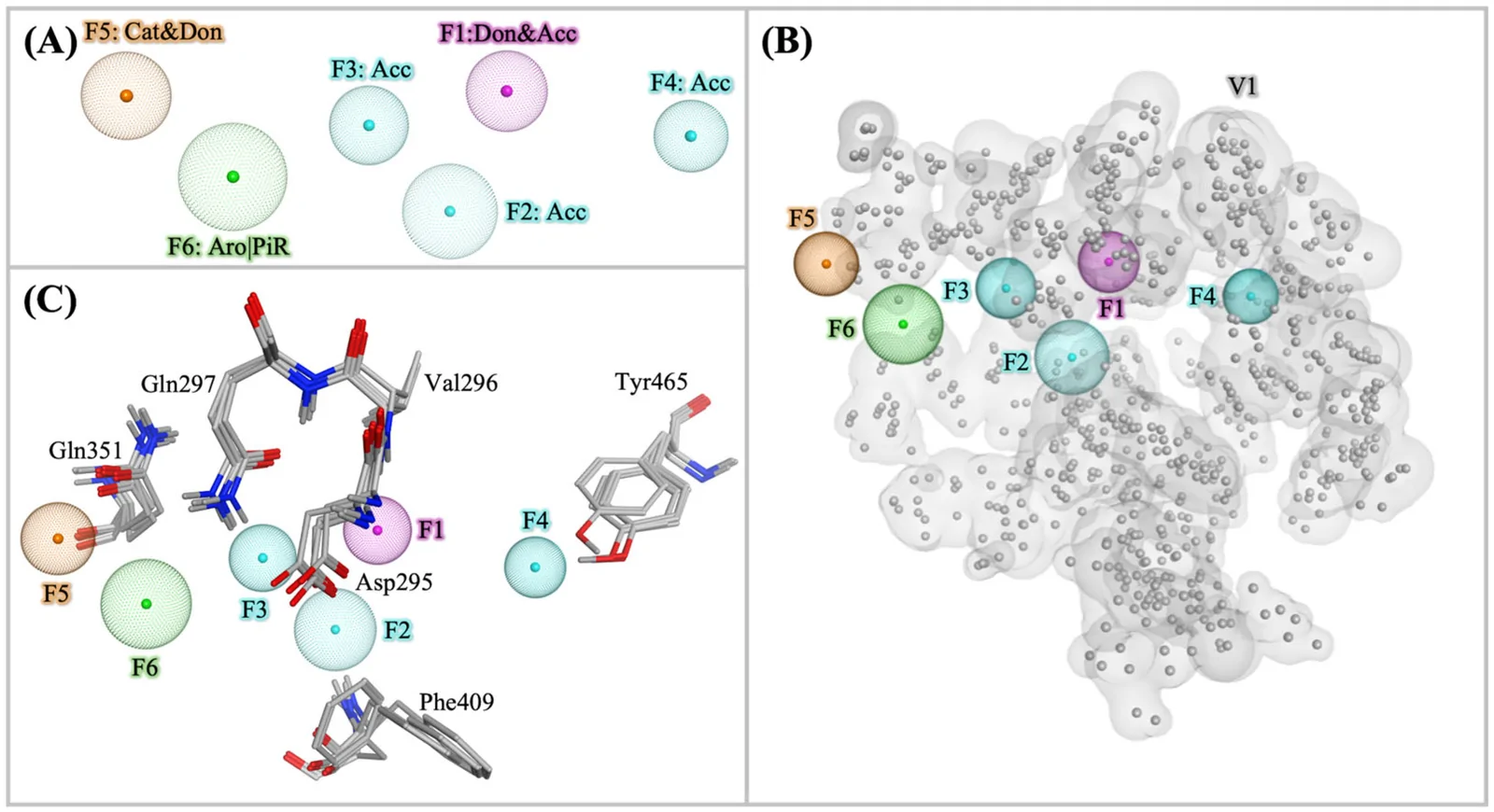
Pharmacophore Model A. (**A**) Pharmacophore model with six features. (**B**) Pharmacophore model with six features and excluded volumes. (**C**) Key residues of USP7 substrate-binding pocket superposed with the pharmacophoric features. F1: Hydrogen bond donor and acceptor (Don & Acc), Radius = 1.1 Å, Magenta; F2: Hydrogen bond acceptor (Acc), Radius = 1.4 Å, Cyan; F3: Hydrogen bond acceptor (Acc), Radius = 1.1 Å, Cyan; F4: Hydrogen bond acceptor (Acc), Radius = 1.0 Å, Cyan; F5: Cation and Hydrogen bond donor (Cat & Don), Radius = 1.1 Å, Orange; F6: Aromatic center or π ring normal (Aro|PiR), Radius = 1.4 Å, Light green; V1: Excluded volumes (ExcV), Radius = 0.9 Å, Grey.

Pharmacophore Model A hypotheses were developed using the AutoPH4 field-based method of the Molecular Operating Environment (MOE) 2022.02 software package (Chemical Computing Group, Montreal, Quebec, Canada) and selected based on protein–ligand interaction fingerprints derived from the PDB entries 5N9T, 5NGE, 5VS6, and 6M1K. The corresponding interactions were analyzed and are summarized in Table S3 (Supporting Information). This pharmacophore model consists of six features (Figure 3). The hydrogen-bond donor/acceptor feature F1 is positioned between the carbonyl side chain of Asp295 and the backbone NH group of Val296. This interaction is conserved across all four crystal structures and is considered critical for USP7 inhibition, as Asp295 directly hydrogen-bonds to the C-terminal region of Ubal and interacts with the amide group of Leu73 [38]. The hydrogen-bond acceptor feature F2 is oriented toward the backbone NH group of Phe409, a residue involved in tunnel formation that acts as a gatekeeper for ubiquitin positioning. Therefore, optimal interaction between a potential small-molecule USP7 inhibitor and this residue appears essential to prevent extension of the ubiquitin tail towards the catalytic triad, thereby promoting USP7 inhibition [38,39]. The hydrogen-bond acceptor feature F3 is located at the vicinity of the backbone NH group of Gln297, an interaction observed in all crystal structures and thus likely to be functionally relevant. In addition, the hydrogen-bond acceptor feature F4, located in the deepest regions of the pocket, represents interactions with the sidechain hydroxyl group of Tyr465. Together with Phe409 and Tyr514, Tyr465 contributes to the tunnel formation and functions as a gatekeeper residue regulating ubiquitin positioning. Its role in ubiquitin recognition further highlights its relevance for USP7 inhibition [38,40]. Finally, one hydrogen-bond donor/cationic feature (F5) and one aromatic feature (F6) were included in the model. F5 reflects interactions with the carbonyl side chain of Gln351, whereas F6 represents π–π interactions between the aromatic moieties of the co-crystallized ligands and Gln351. Notably, Gln351 is solvent-exposed at the entrance of the C-terminal ubiquitin-binding pocket of USP7 and is unique to USP7 among the USP family of protein [33]. USP14 is the only other family member that contains a residue at the equivalent position, whereas most other USPs harbor a residue with a smaller side chain. Therefore, targeting Gln351 may be key to the selectivity of USP7 inhibitors [41].

**Figure 4:**
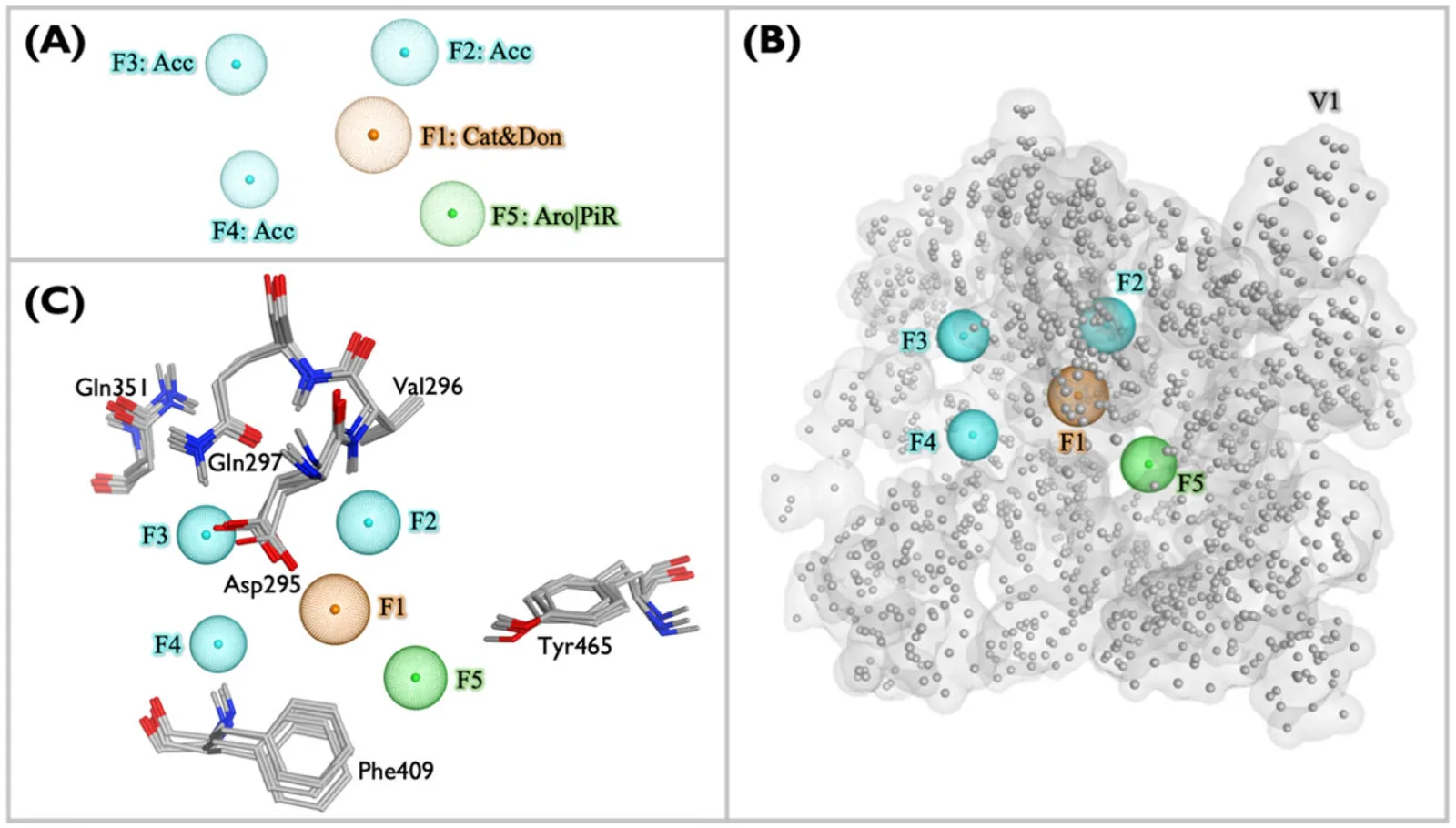
Pharmacophore Model B. (**A**) Pharmacophore model with five features. (**B**) Pharmacophore model with five features and excluded volumes. (**C**) Key residues of USP7 substrate-binding pocket superposed with the pharmacophoric features. F1: Cation and Hydrogen bond donor (Cat & Don), Radius = 1.1 Å, Orange; F2: Hydrogen bond acceptor (Acc), Radius = 1.1 Å, Cyan; F3: Hydrogen bond acceptor (Acc), Radius = 1.0 Å, Cyan; F4: Hydrogen bond acceptor (Acc), Radius = 1.0 Å, Cyan; F5: Aromatic center or π ring normal (Aro|PiR), Radius = 1.1 Å, Light green; V1: Excluded volumes (ExcV), Radius = 0.9 Å, Grey.

Pharmacophore Model B hypotheses were also developed using the AutoPH4 field-based method available in MOE 2022.02 and selected based on the protein–ligand interaction fingerprints from the PDB structures 6VN2, 6VN3, 6VN5 and 6VN6. The corresponding interactions were analyzed and are summarized in Table S3 (Supporting Information). The pharmacophore model consists of five features (Figure 4). The hydrogen-bond donor/cationic feature F1 is oriented toward the carbonyl side chain of Asp295, while the hydrogen-bond acceptor feature F2 is directed toward the backbone NH group of Val296. The hydrogen-bond acceptor feature F3 is located in proximity to the backbone NH group of Gln297. Additionally, one hydrogen-bond acceptor feature (F4) and one aromatic feature (F5) were included in the model. Feature F4 corresponds to interactions with the backbone NH group of Phe409, whereas F5 represents aromatic interactions between the co-crystallized ligands from PDB entries 6VN3 (compound **23**) and 6VN6 (compound **14**) and Phe409, mediated by their benzene and benzofuran moieties, respectively. In agreement with the rationale described for model A, interactions involving the residues Asp295, Val296, Gln297, and Phe409 are proposed to be critical for efficient USP7 inhibition and were therefore incorporated into the pharmacophore model B hypothesis.

For both pharmacophore models, hypotheses containing at least four features were generated. Three features (F1, F2, and F3) were defined as mandatory for all hypotheses in both models, while additional constraints were applied when using the partial match mode. Under these conditions, 21 and 11 pharmacophore hypotheses were generated for models A and B, respectively, each comprising at least four features. Thereafter, the generated pharmacophore hypotheses were validated using multiple statistical metrics, including accuracy, sensitivity, specificity, enrichment, and the power metric, based on a dataset of active compounds and decoys (Table 1).

Analysis of the validation data from the SB-pharmacophore modeling approach indicates that four hypotheses (Table 1), Hyp_A1, Hyp_A2, Hyp_A5, and Hyp_B4 exhibit high selectivity and accuracy (>97%). Among them, Hyp_A5 and Hyp_B4 show the lowest sensitivity (65% and 38%, respectively), although both maintain high specificity (>98%). In contrast, Hyp_A1 and Hyp_A2 combine high sensitivity (>92%) and high specificity (>99%). Hyp_A1 demonstrated more than 45-fold enrichment over random in identifying active compounds from chemical databases. In addition, all models achieved greater than 50-fold enrichment in the top-ranked subset (EF_0.5%_). Therefore, based on the validation results, the four pharmacophore hypotheses summarized in Table 2 were selected as the 3D screening queries to enable efficient compound prioritization for downstream molecular docking and hit identification.

### 2.3. Virtual Screening and Molecular Docking

The docking protocol was validated through self-docking experiments to evaluate the performance of scoring functions available in MOE 2022.02. The best performance was obtained using London dG for placement and GBVI/WSA dG for refinement. This protocol accurately reproduced the binding pose of the co-crystallized ligand (L55) in the USP7 structure (PDB ID: 6M1K), yielding a root mean square deviation (RMSD) of 0.68 Å and a docking score of −11.76 (Figure S3, Supporting Information). The obtained RMSD value supports the suitability of the protocol for ligand pose prediction.

To further evaluate the ability of the docking protocol to discriminate active from inactive compounds, enrichment validation was performed using the combined benchmark dataset previously generated for pharmacophore validation, comprising 55 experimentally validated USP7 inhibitors and 3340 property-matched DUD-E decoys. The protocol achieved enrichment factors of 44, 36 and 10 at the top 0.5%, 1% and 5% of the ranked database, respectively (EF_0.5%_ = 44, EF_1%_ = 36, and EF_5%_ = 10), demonstrating good early enrichment of known USP7 inhibitors over property-matched decoys and further supporting the suitability of the docking protocol for prospective virtual screening. The previously prepared database of approximately 500,000 unique compounds was screened using all four validated pharmacophore models, yielding 25,456 compounds matching the defined pharmacophoric features. These compounds were subsequently docked into the USP7 inhibitor-binding site of the PDB entry 6M1K using MOE 2022.02. Following docking, compounds were ranked according to their docking scores. The 500 highest-ranked compounds were then subjected to detailed visual inspection, and their predicted protein–ligand interactions were systematically analyzed using the Protein-Ligand Interaction Profiler (PLIP). Compound prioritization for in vitro assays further considered the presence of key hydrogen-bond interactions with catalytically or structurally relevant residues, favorable hydrophobic and π–π interactions within the binding pocket, occupancy of computationally identified hot spot regions, and the absence of significant steric clashes or energetically unfavorable conformations. Structural diversity, compound availability, and synthetic accessibility were also considered. Based on these criteria, 35 compounds were selected for experimental validation, including 5 from the National Cancer Institute (NCI) compound database, 9 from the MedChemExpress (MCE) libraries, and 21 from the in-house collection.

### 2.4. Primary Screening Identifies Candidate USP7-Inhibitory Scaffolds

**Figure 5:**
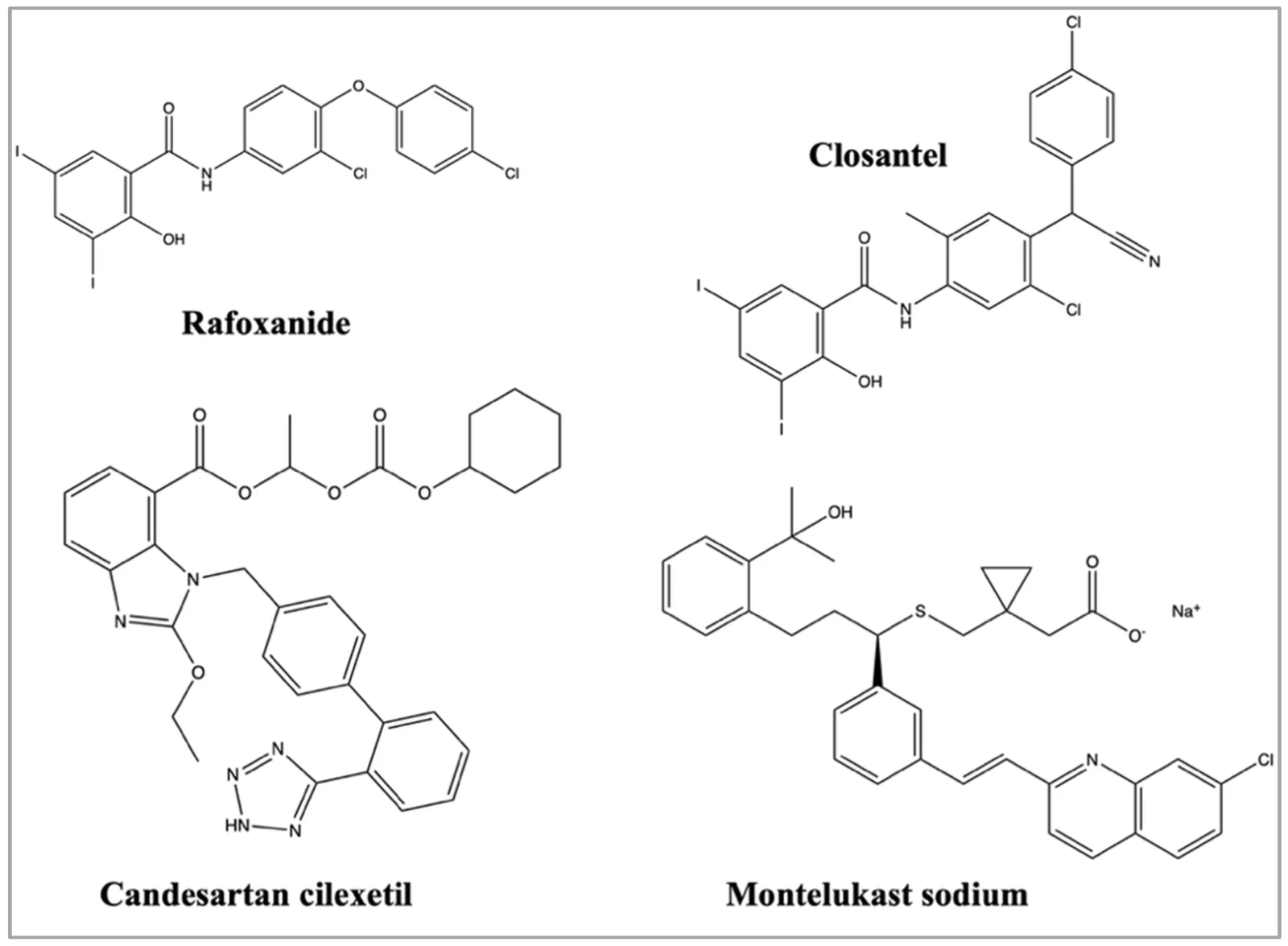
Chemical structures of the four compounds identified herein as USP7 hit inhibitors.

To assess the predictive performance of the in silico workflow, the selected compounds were evaluated for their inhibitory activity against USP7 in vitro, using recombinant full-length USP7 (USP7-FL) and the fluorogenic substrate ubiquitin-rhodamine 110 (Ub-Rho110). Based on their inhibitory activity at 10 µM, compounds were classified as active (>50% inhibition), weakly active (10–49% inhibition), or inactive (<10% inhibition). The primary screening identified four active compounds and twenty-one weakly active compounds, including structurally diverse approved drugs and compounds from the in-house chemical library (Table 3 and Table S4, Supporting Information). The four active screened compounds (Figure 5) were subsequently selected for dose–response analysis against both USP7-FL and the USP7 catalytic domain (USP7-CD). Inhibitory potency was determined using six-point dose–response experiments performed in triplicate (Table 4 and Table S5, Figure S4, Supporting Information). All four compounds exhibited micromolar inhibitory activity against USP7 and consistently displayed greater potency toward USP7-CD than USP7-FL, likely reflecting the greater conformational accessibility of the isolated catalytic domain.

To evaluate the potential influence of assay interference, additional control experiments were performed at the highest concentration used in the dose–response assays (100 μM). Intrinsic compound fluorescence and the fluorescence of intact Ub-Rho110 were assessed under the same excitation and emission settings used for the USP7 inhibition assay. Montelukast sodium and candesartan cilexetil exhibited detectable intrinsic fluorescence in assay buffer, whereas closantel and rafoxanide displayed comparatively lower fluorescence signals. In the presence of intact Ub-Rho110, fluorescence signals remained comparable to the vehicle control for montelukast sodium and candesartan cilexetil, whereas lower fluorescence intensities were observed for closantel and rafoxanide (Figure S5, Supporting Information). Following the generation of Rho110 by USP7-mediated substrate cleavage, fluorescence measurements showed minimal changes for montelukast sodium and candesartan cilexetil. Meanwhile, closantel and rafoxanide modestly reduced the fluorescence signal by around 20% (Figure S6, Supporting Information). In addition, increasing the Tween-20 concentration ten-fold (0.002% to 0.02%) did not appreciably affect the inhibitory activity of any of the tested compounds (Figure S7, Supporting Information).

### 2.5. Molecular Docking Analysis and In Silico ADME Profiling of the Candidate USP7-Inhibitory Scaffolds

**Figure 6:**
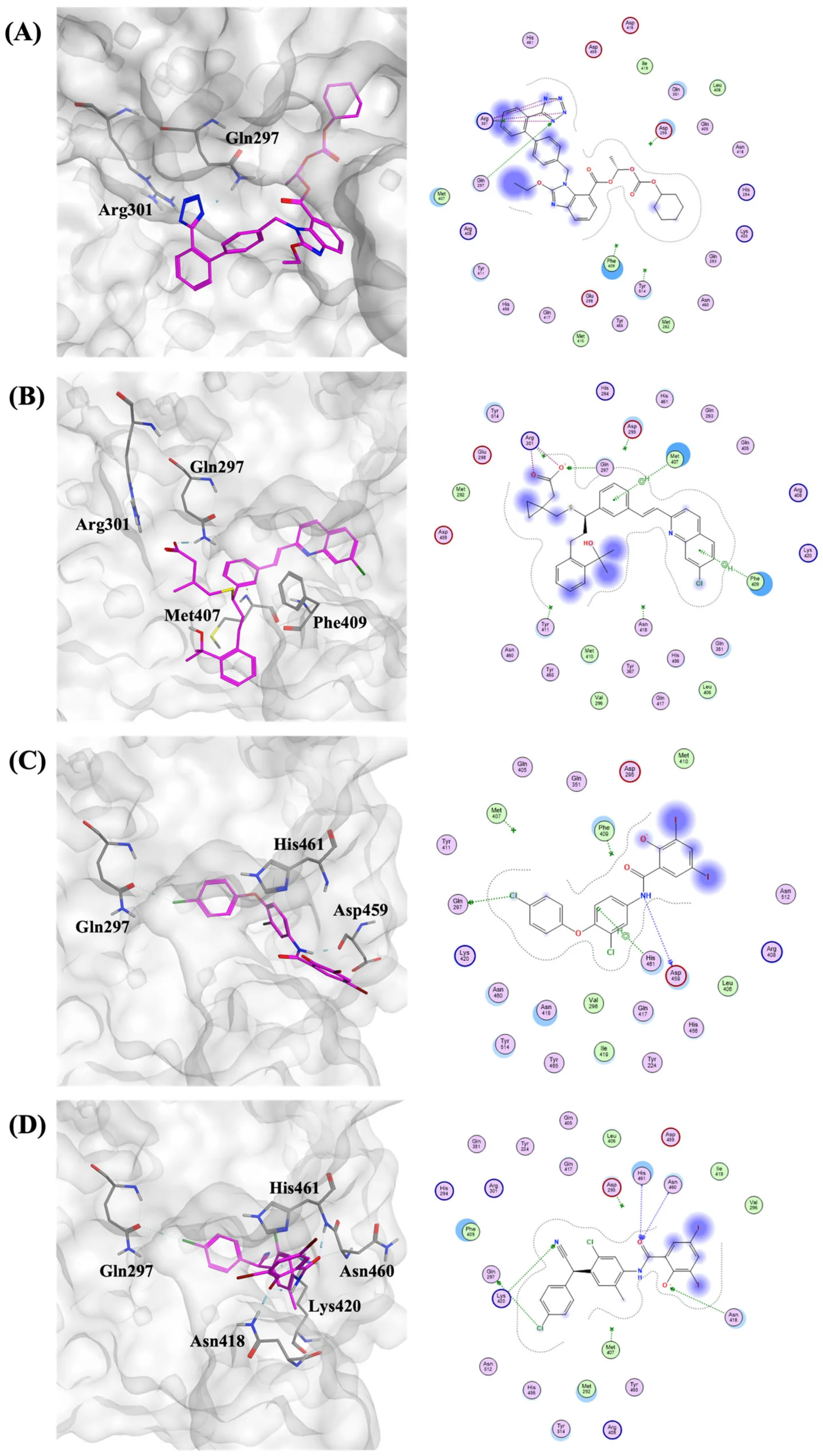
Predicted binding modes and protein–ligand interaction profiles of the four candidate USP7-inhibitory scaffolds docked into the USP7 active site (PDB ID: 6M1K). (**A**) candesartan cilexetil, (**B**) montelukast sodium, (**C**) rafoxanide, and (**D**) closantel. The left-hand panels show the binding poses within the USP7 active site, while the right-hand panels depict the corresponding 2D diagrams of the ligand–protein interactions. Interacting residues are highlighted. Arrows indicate hydrogen-bond interactions (green: side chain donor/acceptor; blue: backbone donor); dashed green lines with an arene symbol denote π–hydrogen interactions; red dashed lines indicate electrostatic interactions. Residues are colored by type: hydrophobic (green) and polar (purple), with blue and red contours representing basic and acidic residues, respectively. Figures were generated using MOE 2022.02.

Analysis of the predicted binding modes of the four candidate USP7-inhibitory scaffolds suggests that ligand recognition within the USP7 binding site is primarily mediated by hydrogen-bonding interactions with Gln297, Asn418, Lys420, Asp459, Asn460, and His461, complemented by electrostatic interactions with Arg301 and C–H···π interactions with Phe409 (Table 5 and Figure 6).

In candesartan cilexetil, the tetrazole ring engages in multiple electrostatic interactions with the positively charged guanidinium group of Arg301 (~3 Å), while one of its tetrazole nitrogen atoms forms a hydrogen bond with the side-chain amide of Gln297 (3.65 Å). Similarly, in montelukast sodium, the terminal carboxyl group establishes electrostatic interactions with Arg301 (~3.72 Å) and forms a hydrogen bond with Gln297 (2.95 Å). In addition, montelukast sodium participates in two C–H···π interactions: one involving its benzene ring and the backbone of Met407 (4.30 Å), and another between its chlorobenzene ring (from the chloroquinoline moiety) and the side chain of Phe409 (4.74 Å). In rafoxanide, the chlorine atom on the lateral benzene ring engages in a halogen bond with the side-chain carbonyl of Gln297 (3.29 Å). An additional hydrogen bond is formed between the ligand amine and the backbone carbonyl of Asp459 (3.01 Å), and a C–H···π interaction is observed with the imidazole ring of His461 (3.49 Å). In closantel, the chlorine atom on the lateral benzene ring similarly forms a halogen bond with the side-chain carbonyl of Gln297 (3.50 Å). The nitrile nitrogen acts as a hydrogen-bond acceptor in a N···C–H interaction with the side chain of Lys420 (3.19 Å). The amide carbonyl forms a hydrogen bond with the backbone amine of His461 (2.98 Å) and a weaker C–H···O interaction with the backbone of Asn460 (3.08 Å). Finally, the benzylic hydroxyl group establishes a hydrogen bond with the side-chain amide nitrogen of Asn418 (2.97 Å).

Overall, the predicted binding modes are consistent with known USP7 inhibitor-binding hotspots, particularly involving Gln297, His461, Asp459, and Phe409, which have been frequently implicated in ligand recognition and stabilization within the catalytic pocket. Additional interactions with peripheral residues such as Asn418 and Lys420 further contribute to ligand positioning. Electrostatic contacts observed with Arg301 appear to be ligand-specific and may reflect local structural adaptations rather than a conserved hotspot. To complement the binding-mode analysis of the selected hits, their pharmacokinetic properties were subsequently evaluated using in silico ADME prediction tools.

According to the in silico prediction performed in SwissADME [42], the identified candidate USP7-inhibitory scaffolds generally exhibited high lipophilicity (consensus LogP_o/w_ > 5), with the exception of candesartan cilexetil (LogP_o/w_ = 4.46), and were predicted to possess low aqueous solubility (LogS = −7.66 to −8.60) (Table 6). Candesartan cilexetil and montelukast sodium were also predicted to inhibit CYP3A4, suggesting a potential risk of metabolic liabilities and drug–drug interactions. Overall, these predictions indicate that the selected compounds possess physicochemical and pharmacokinetic properties that may require further optimization, particularly with respect to aqueous solubility and metabolic profile. Nevertheless, the combined docking and ADME analyses supported the selection of these compounds for subsequent in vitro evaluation of their antiproliferative activity.

### 2.6. In Vitro Antiproliferative Activity in Human Cancer and Non-Tumorigenic Cells

To evaluate the antiproliferative activity of the validated candidate USP7-inhibitory scaffolds, the compounds were subsequently tested in vitro across a panel of breast, lung, and pancreatic cancer cell lines characterized by distinct molecular backgrounds (Table 7).

Montelukast sodium and candesartan cilexetil displayed relatively weak to moderate activity, with IC_50_ values generally in the range of 45 to 90 µM across all tested cell lines. In contrast, closantel demonstrated improved activity, with IC_50_ values ranging from 20 to 52 µM, indicating moderate potency and a more consistent antiproliferative effect across the different cancer types. Notably, enhanced activity was observed in pancreatic cancer cell lines, where IC_50_ values were as low as 20 µM.

Among all evaluated compounds, rafoxanide displayed the greatest antiproliferative potency, consistently exhibiting low- to mid-micromolar activity across all tested cancer cell lines. The IC_50_ values ranged from 11 to 33 µM in lung and breast cancer cells and from 22 to 24 µM in pancreatic cancer cell lines. The lowest IC_50_ value (11 µM) was observed in the H1437 lung cancer cell line.

To obtain a preliminary assessment of selectivity, the compounds were also evaluated in the non-tumorigenic human mammary epithelial cell line MCF10A. Rafoxanide consistently exhibited lower growth-inhibitory effects in MCF10A cells than in the cancer cell lines, resulting in favorable selectivity index (SI) values and suggesting a preliminary selectivity profile toward malignant cells (Table 8).

To facilitate comparison of the validated hit compounds, Table 9 summarizes their biochemical activity against USP7, antiproliferative potency, preliminary selectivity, predicted physicochemical properties, and key optimization priorities. Overall, rafoxanide demonstrated the most favorable combination of biochemical activity against USP7, antiproliferative potency, and a favorable preliminary selectivity profile toward malignant cells among the compounds evaluated, supporting its prioritization for further hit-to-lead optimization and mechanistic validation.

## 3. Discussion

The present study integrated pharmacophore modelling, structure-based virtual screening, molecular docking, in silico pharmacokinetic profiling, and in vitro biological evaluation to identify candidate USP7-inhibitory scaffolds exhibiting antiproliferative activity.

Importantly, the structure-based virtual screening workflow was intentionally performed without prior application of Lipinski’s rule of 5 (Ro5) to the screening library. Although Ro5 and related property-based criteria are routinely employed during virtual screening campaigns, these parameters were originally developed as empirical guidelines for oral bioavailability rather than strict exclusion criteria for early-stage hit discovery. Indeed, a significant proportion of approved drugs violate at least one Ro5 parameter, and the number of clinically successful compounds occupying the beyond Ro5 (bRo5) chemical space has steadily increased, particularly among scaffolds targeting challenging binding sites such as protein–protein interaction interfaces [43,44]. Therefore, rigid pre-docking filtering may inadvertently exclude chemically diverse and pharmacologically relevant chemotypes at the earliest stages of discovery. Since potency and target engagement are often more difficult to optimize than physicochemical properties, we prioritized broad exploration of the chemical space during hit identification and deferred pharmacokinetic optimization to subsequent stages of lead refinement [45,46]. This strategy enabled the identification of structurally diverse candidate USP7-inhibitory scaffolds that would have been eliminated by conventional filtering approaches.

Four candidate USP7-inhibitory scaffolds exhibiting micromolar inhibitory activity were selected from the virtual screening workflow, all of which exhibited greater potency against the isolated catalytic domain than against the full-length enzyme. These differences in activity likely reflect the structural and conformational complexity governing ligand engagement in USP7. USP7 is a multidomain deubiquitinase whose catalytic activity is dynamically regulated by the N-terminal TRAF-like domain and the C-terminal ubiquitin-like (UBL) domains through intramolecular interactions and conformational rearrangements [14,15,16]. The enhanced potency observed against USP7-CD suggests that removal of the enzyme’s regulatory regions increases accessibility to the catalytic cleft and alleviates conformational constraints that limit ligand binding in the full-length enzyme. In agreement with previous structural studies, the reduced activity observed for USP7-FL likely reflects a more conformationally restricted and physiologically relevant enzymatic state [16,47]. These findings underscore the importance of evaluating candidate USP7-inhibitory scaffolds in the context of the full-length enzyme, as assays relying exclusively on isolated catalytic domains may overestimate inhibitor potency and fail to capture regulatory effects imposed by the native domain architecture. Nevertheless, detailed structural and biophysical characterization of the conformational differences between USP7-CD and USP7-FL was beyond the scope of the present study.

Given the susceptibility of fluorescence-based enzymatic assays to optical and other assay interferences, the inhibitory activity of the selected compounds was further examined through a series of control experiments designed to assess intrinsic fluorescence, potential effects on substrate and product fluorescence, and detergent sensitivity. Although montelukast sodium and candesartan cilexetil exhibited intrinsic fluorescence, and closantel and rafoxanide produced a modest reduction in the fluorescence signal of the liberated Rho110 fluorophore, these effects were small relative to the observed enzymatic inhibition and are therefore unlikely to fully account for the measured USP7 inhibitory activity. Because fluorescence quenching was assessed only at the highest tested concentration (100 μM), these measurements were intended to identify the potential contribution of optical interference rather than to provide a concentration-dependent correction of the enzymatic dose–response data and therefore do not constitute an appropriate basis for quantitively correcting the reported inhibition values or IC_50_ estimates. Likewise, the absence of any appreciable change in inhibitory activity upon a ten-fold increase in Tween-20 concentration argues against detergent-sensitive colloidal aggregation as the primary mechanism underlying the observed inhibition. Collectively, these control experiments strengthen confidence that the observed USP7 inhibition is not predominantly driven by fluorescence-related artifacts or detergent-sensitive aggregation under the assay conditions employed. However, as with any fluorescence-based screening assay, these controls do not establish direct target engagement or define the molecular mechanism of inhibition.

The present study was designed as an early-stage hit-identification campaign rather than a comprehensive mechanistic characterization. Consequently, orthogonal validation and selectivity profiling were intentionally reserved for subsequent hit-to-lead optimization. Future studies will therefore employ complementary biochemical and biophysical approaches, including orthogonal enzymatic assays, activity-based probe labeling, direct binding methods, and cellular target-engagement assays monitoring canonical USP7 substrates, to confirm direct USP7 engagement and elucidate the inhibitory mechanism of the identified scaffolds. In addition, the selectivity of the identified compounds toward USP7 relative to other deubiquitinases was not investigated in the present study. Given the structural and mechanistic similarities among DUB family members, future work will include profiling against closely related USPs, such as USP47, together with representative members of other DUB families, to establish enzyme selectivity, assess potential off-target inhibition, and determine whether the observed cellular phenotypes are mediated specifically through USP7 inhibition. Docking studies predicted that all selected compounds occupy the USP7 catalytic cleft and engage a conserved interaction network involving residues previously implicated in inhibitor recognition, including Gln297, Met407, Phe409, Asp459, Asn460, and His461 [33,41]. These residues are known to contribute to the stabilization of USP7–inhibitor complexes in co-crystal structures of established inhibitors such as FT671 and FT827, which inhibit USP7 by stabilizing inactive conformational states of the catalytic domain [33]. The consistency between the predicted binding modes and the patterns of previously reported protein-ligand interactions provides structural support for the proposed binding hypotheses. Collectively, these observations suggest that the identified scaffolds engage a pharmacologically relevant region of the enzyme. Among these interactions, Gln297 emerged as a key anchoring residue, forming conserved hydrogen-bonding or polar interactions across all evaluated compounds. This observation further supports Gln297 engagement as a critical determinant of ligand positioning within the USP7 catalytic pocket [33,41]. By contrast, electrostatic interactions involving Arg301 were observed only in selected docking poses. Although Arg301 is located at the periphery of the USP7 inhibitor-binding region, it is not among the residues most frequently reported to mediate ligand recognition in available USP7 co-crystal structures [33,41]. These observations suggest that Arg301-mediated contacts may contribute to ligand-specific binding modes rather than representing a universally required determinant for USP7 inhibition. Notably, rafoxanide displayed one of the most favorable predicted interaction profiles within the catalytic cleft, forming multiple stabilizing contacts that may partially account for its comparatively higher biochemical inhibitory activity and effects among the selected hit compounds. Nevertheless, the proposed binding modes should be regarded as plausible binding hypotheses rather than definitive representations of the protein–ligand complexes. Future studies employing molecular dynamics simulations, together with complementary structural and biophysical approaches, will be valuable for assessing the stability of the predicted binding poses, exploring alternative protein conformations, and validating the proposed ligand–protein interactions.

Although the identified compounds inhibited recombinant USP7 and exhibited antiproliferative activity across multiple cancer cell lines, the present study does not establish that the observed cellular effects are directly mediated by USP7 inhibition. Consequently, the antiproliferative activity should be interpreted as a biological property of compounds that also exhibit biochemical USP7 inhibitory activity, rather than as definitive evidence of USP7-driven anticancer effects. Future studies will focus on confirming cellular target engagement through the evaluation of USP7-regulated signaling pathways and relevant downstream biomarkers, together with genetic validation approaches, such as USP7 knockdown, overexpression, or rescue experiments, to determine the extent to which the observed antiproliferative activity is mediated by USP7 inhibition. Furthermore, although several of the identified compounds are approved drugs with well-characterized pharmacological and safety profiles, their physicochemical characteristics present important limitations for development as USP7-targeted anticancer agents. In particular, their high molecular weights, elevated lipophilicity, multiple Lipinski rule violations, and poor predicted aqueous solubility are expected to adversely affect solubility, formulation, and pharmacokinetic behavior. Consequently, the identified compounds should be regarded as early-stage candidate USP7-inhibitory scaffolds that expand the chemical space available for future medicinal chemistry optimization rather than as optimized lead compounds.

## 4. Materials and Methods

### 4.1. Dataset Preparation

To enable comprehensive yet chemically diverse exploration of candidate molecules, four complementary compound libraries were selected for virtual screening: the NCI compound database, the MCE FDA-approved drug library plus, the MCE bioactive compound library, and an in-house collection of natural and semi-synthetic derivatives. Following curation and removal of duplicates, the final screening database comprised approximately 500,000 unique compounds. The integration of large public repositories with a curated in-house collection maximized structural diversity while maintaining chemical relevance.

Since experimentally validated USP7 inactive compounds (decoys) were not present in public benchmarking databases as of November 2022, a validation dataset comprising active compounds and decoys was manually curated. Active compounds with experimentally determined USP7 IC_50_ values below 1 μM were collected from the literature [48,49,50,51,52]. Subsequently, the ligand structures in SMILES format were submitted to the Database of Useful Decoys-Enhanced (DUD-E) web tool (http://dude.docking.org/generate, accessed on 13 November 2022) [53]. Decoys with similar physicochemical properties but minimized topological similarity to the active compounds were retrieved from the ZINC database [54], yielding approximately 50 decoys per active ligand. The active compounds represented the experimentally characterized pyrazolopyrimidinone- and pyridylbenzofuran-based USP7 inhibitor series available at the time of dataset construction. The final validation datasets consisted of 26 active ligands and 1292 decoys for pharmacophore Model A, and 29 active ligands and 2048 decoys for pharmacophore Model B. Duplicate entries were removed from both the active and decoy datasets during curation to avoid overrepresentation of identical compounds. No further similarity filtering was applied prior to pharmacophore validation.

All compound datasets were prepared using MOE 2022.02. Ligands in SMILES format were converted into MOE molecular database (.mdb) files, followed by structure preparation using the Wash module at physiological pH (7.4). Hydrogen atoms were added and protonation states assigned according to the dominant species at pH 7.4, while the stereochemistry and input tautomeric forms of the original databases were retained. Energy minimization was performed using the AMBER10:EHT force field with the Born solvation model. Conformational ensembles were then generated using the Conformation Import tool, applying a strain energy cutoff of 4.0 kcal/mol and a maximum limit of 500 conformations per molecule. Finally, partial atomic charges were calculated, and hydrogen atoms and lone pairs were optimized accordingly.

### 4.2. Protein Structure Preparation

Eight USP7-ligand complexes were retrieved from the Research Collaboratory for Structural Bioinformatics (RCSB) Protein Data Bank (PDB) under the following PDB identifiers: 5N9T, 5NGE, 5VS6, 6M1K, 6VN2, 6VN3, 6VN5 and 6VN6. For each complex, only receptor chain A and the corresponding co-crystallized ligand were retained, while all crystallographic water molecules and other non-essential heteroatoms were removed. Protein preparation was performed using the structure preparation tool of MOE 2022.02, and protein structural issues corrected with the addition of missing atoms and residues (if any), bond order, formal charge correction, and tautomer adjustment. Using protonate three-dimensional (3D), the structures were protonated (at pH 7.4 and 310.15 K), followed by the addition of hydrogen atoms. The Amber10:EHT force field with the solvation model set to Born was used to assign atom types and partial charges to each atom in the receptor structure. The resulting protein structures were subsequently energy minimized using the same force field to relieve local steric clashes while preserving experimental geometry. Finally, all USP7 crystal structures were structurally aligned and superposed using the MOE Sequence Editor to enable comparative analysis and pharmacophore model generation [55,56,57].

### 4.3. Binding Hot Spot Identification Using FTMap

Potential druggable hot spots within the USP7 catalytic domain were identified using the computational mapping server FTMap (accessed 24 November 2022). FTMap is a fragment-based mapping approach designed to identify energetically favorable binding regions on protein surfaces for small drug-like molecules [58]. Four USP7 crystal structures (PDB IDs: 5N9T, 6M1K, 6VN2 and 6VN6) obtained from the RCSB Protein Data Bank were submitted to the FTMap server in PDB format. The FTMap algorithm performs exhaustive sampling of billions of binding poses for a panel of 16 small organic probe molecules with varying sizes, shapes, and polarities across both surface-exposed and buried regions of the protein. Probe conformations were subsequently energy-minimized, clustered, and ranked according to their Poisson–Boltzmann averaged interaction energies. Regions where multiple probe clusters converged spatially were defined as CSs. The CSs containing the highest number of probe clusters were classified as main hot spots, whereas the remaining sites were considered secondary hot spots. To identify conserved ligand-binding regions, the CSs obtained from the different USP7 crystal structures were structurally aligned and comparatively analyzed to determine spatially concordant hot spots across the ensemble of receptor conformations.

A major application of FTMap is the assessment of protein druggability through the identification of energetically favorable regions for small-molecule binding. According to the FTMap methodology, a binding site is considered potentially druggable when it contains a dominant CS comprising more than 16 probe clusters, indicative of a strong main hot spot. In contrast, sites containing 13–16 probe clusters are classified as weak hot spots with uncertain druggability, as they may support only low-micromolar ligand binding and are generally less amenable to affinity optimization. Additionally, druggable regions typically require at least one neighboring secondary hot spot located within 8 Å of the main hot spot, thereby enabling cooperative ligand–protein interactions that favor high-affinity binding [58].

As a computational surrogate to the costly and time-consuming X-ray crystallography and Nuclear Magnetic Resonance (NMR)-based screening experiments, FTMap identifies potential ligand-binding hot spots by systematically mapping small organic probe molecules onto the protein surface and evaluating their interaction propensities [58,59]. For each PDB structure, the server calculates non-bonded and hydrogen-bond interactions between probes and protein residues using HBPlus. Residue contact frequency can be expressed as the percent contact frequency, defined as the number of interactions formed by a given residue divided by the total number of interactions observed across all residues for a given interaction type [58].

### 4.4. Pharmacophore Generation and Pharmacophore-Based Virtual Screening

At the time of this study (November 2022), the PDB contained USP7 crystal structures co-crystallized with ligands targeting the ubiquitin C-terminal tail-binding pocket (Ubal) that could be broadly classified into two chemically distinct scaffold families: pyrazolopyrimidinone-based and pyridylbenzofuran-based inhibitors. Two independent structure-based (SB) pharmacophore models were generated: Model A, derived from pyrazolopyrimidinone-based inhibitors, and Model B, derived from pyridylbenzofuran-based inhibitors.

The SB pharmacophore models were constructed from experimentally resolved USP7–ligand complexes retrieved from the PDB. Four crystal structures were selected for each model based on ligand scaffold similarity and binding mode conservation. Model A was generated using PDB IDs 5N9T, 5NGE, 5VS6, and 6M1K, whereas Model B was derived from PDB IDs 6VN2, 6VN3, 6VN5, and 6VN6. Pharmacophoric annotation points from both receptor and ligand environments were extracted as features using the AutoPH4 field-based methodology implemented in MOE 2022.02. Feature selection was subsequently refined according to the conserved protein–ligand interaction fingerprints observed across the aligned co-crystal structures [60]. Subsequently, the generated pharmacophoric features were manually refined using the unified annotation scheme implemented in the MOE Pharmacophore Editor module. To further support pharmacophore construction and prioritize energetically relevant interaction regions, the computational mapping data obtained from FTMap was integrated into the model refinement process. Six and five final pharmacophoric features were retained for Models A and B, respectively. Multiple pharmacophore hypotheses comprising different feature combinations were subsequently generated and subjected to retrospective validation against the curated active/decoy datasets. The four hypotheses exhibiting the best discriminatory performance were selected for subsequent pharmacophore-based virtual screening. The selected pharmacophore hypotheses were then used as three-dimensional structural queries for virtual screening of the prepared chemical libraries using the Pharmacophore Search module implemented in MOE 2022.02. Depending on the specific query, either total-match or partial-match search modes were applied.

### 4.5. Pharmacophore Model Validation

The predictive performance of the pharmacophore models was assessed using multiple statistical metrics. Retrospective validation was conducted using a dataset composed of literature-reported USP7 inhibitors and decoys generated with the DUD-E web tool. Model accuracy (Equation (1)) was defined as the proportion of compounds correctly classified as active or inactive and was calculated by

(1) $$\%Accuracy=\frac{TP+TN}{D}$$

where true positive (TP) is the number of USP7 inhibitors correctly identified as active, true negative (TN) is the number of non-USP7 inhibitors correctly identified as inactive and D is the total number of compounds in the validation set.

Sensitivity (Se), also referred to as the true positive rate (TPR) (Equation (2)), measures the ability of the model to correctly identify active compounds among all actives in the screening set. Specificity (Sp) (Equation (3)) reflects the ability of the model to correctly classify inactive compounds among all inactives in the dataset, and were calculated by

(2) $$\%Se=TPR=\frac{TP}{TP+FN}$$

(3) $$\%Sp=\frac{TN}{TN+FP}$$

where false negative (FN) is the number of USP7 inhibitors incorrectly identified as inactive and false positive (FP) is the number of non-USP7 inhibitors incorrectly identified as actives.

The false positive rate (FPR) (Equation (4)), complementary to the TPR, is defined as the proportion of inactive compounds misclassified as active among all inactive compounds in the dataset and was calculated by

(4) $$FPR=\frac{FP}{FP+TN}$$

The retrieved compounds in the validation set were ranked according to the RMSD between the pharmacophore query features and the corresponding mapped features of each molecule. The resulting ranked lists were used to calculate the enrichment factor (EF) (Equation 5), which quantifies the fold-enrichment of active compounds among the top-ranked subset relative to random selection. Since early recognition in the ranked list is of particular interest, enrichment factors at 0.5% and 5% of the screened dataset (EF_0.5%_ and EF_5%_, respectively) were also calculated (Equations (6) and (7)) to assess early enrichment. An EF value greater than 1.0 indicates performance better than random selection, reflecting enrichment of active compounds [61,62].

(5) $$EF=\frac{TP\times D}{Ht\times A}$$

(6) $${EF}_{0.5\%}=\frac{{TP}_{0.5\%}\times D}{{Ht}_{0.5\%}\times A}$$

(7) $${EF}_{5\%}=\frac{{TP}_{5\%}\times D}{{Ht}_{5\%}\times A}$$

where A is the total number of active compounds in the dataset, TP_0.5%_ is the number of active compounds in the 0.5% of the ranked dataset, TP_5%_ is the number of active compounds in the 5% of the ranked dataset, Ht is the total number of compounds retrieved by the model, H_t0.5%_ is the number of compounds in the 0.5% of the dataset and Ht_5%_ is the number of compounds in the 5% of the dataset.

The Se and Sp are useful indicators of model performance but do not fully capture predictive power. Therefore, a statistically robust metric, termed the power metric (PM) (Equation (8)), was employed. This metric is relatively insensitive to variations in the proportion of active compounds in the dataset while remaining sensitive to changes in model quality. High-quality models typically yield PM values between 0.9 and 1.0 [63].

(8) $$PM=\frac{TPR}{TPR+FPR}$$

### 4.6. Molecular Docking

The molecular docking simulations were performed using MOE 2022.02 software package [64]. All ligands were prepared as described in the Section 4.1 using the MOE Wash module at physiological pH (7.4), with protonation states assigned accordingly while preserving the stereochemistry and tautomeric states provided in the original databases. The crystal structure of the human USP7 co-crystallized with the inhibitor L55 (PDB ID: 6M1K) was selected as the receptor model and prepared as previously described. The docking binding site was defined as the ensemble of alpha spheres located within a 5 Å radius of the co-crystallized ligand.

It is well established that docking scoring functions display target- and protein family-dependent performance, and therefore no universal scoring function consistently provides optimal predictive accuracy across different systems [65]. Accordingly, the docking protocol was systematically optimized and validated through self-docking experiments using the co-crystallized L55 ligand from PDB entry 6M1K. All scoring functions available in MOE 2022.02 were evaluated based on their ability to reproduce the experimental binding pose. Based on the benchmarking results, the Triangle Matcher placement method combined with the London dG scoring function was selected for initial ligand placement, followed by pose refinement using the GBVI/WSA dG scoring function under a rigid receptor protocol. Under these optimized conditions, the experimentally observed crystallographic pose of L55 was accurately reproduced, yielding a RMSD below the commonly accepted 2.0 Å reliability threshold for docking pose prediction [66,67].

To further assess the ability of the docking protocol to discriminate active from inactive compounds, enrichment validation was performed using the combined benchmark dataset previously generated for pharmacophore validation, comprising 55 experimentally validated USP7 inhibitors and 3340 property-matched DUD-E decoys. All compounds were docked using the optimized protocol described above. The resulting docking output was ranked according to the final docking scores, and early recognition performance was evaluated by calculating the enrichment factors at the top 0.5%, 1%, and 5% of the ranked database (EF_0.5%_, EF_1%_, and EF_5%_).

The validated docking workflow was subsequently applied to the curated virtual screening database comprising approximately 500,000 unique compounds. During ligand placement, 30 poses per compound were generated using the Triangle Matcher algorithm and initially ranked with the London dG scoring function. The resulting poses were then refined and rescored using the GBVI/WSA dG scoring function using a rigid receptor protocol. The five highest-ranked poses for each compound were retained for further analysis, whereas all remaining docking parameters were kept at their default values. For each compound, the most plausible binding pose was selected after comparative evaluation of the five highest-ranked docking solutions, considering both the docking score and the predicted protein–ligand interaction network characterized using PLIP. The 500 highest-ranked compounds were subsequently subjected to detailed visual inspection to assess the plausibility and consistency of the selected binding poses within the USP7 catalytic pocket. Compound prioritization considered the establishment of key hydrogen-bond interactions with catalytically or structurally relevant residues, favorable hydrophobic and π–π interactions within the binding pocket, occupancy of computationally identified hot spot regions, and the absence of significant steric clashes or energetically unfavorable conformations. Compounds satisfying these criteria were prioritized for subsequent experimental evaluation.

### 4.7. Compounds

A library comprising approximately 500,000 compounds was assembled from the NCI compound database, the MCE FDA-approved drug library plus, the MCE bioactive compound library, and an in-house collection of drug-like natural products and semi-synthetic derivatives [68,69,70,71,72,73,74,75]. This curated dataset was used for both pharmacophore-based virtual screening and molecular docking studies. The reference USP7 inhibitor XL188 [41] (Sigma-Aldrich, Darmstadt, Germany, #SML3043) was used as the positive control. Based on the virtual screening workflow, 35 compounds were selected for in vitro validation using freshly prepared stock solutions. Of these, nine compounds were obtained from commercial suppliers, five were sourced from the NCI repository, and the remaining 21 compounds originated from the in-house chemical collection. Candesartan cilexetil (98% purity, CAS No. 145040-37-5), closantel (99% purity, CAS No. 57808-65-8), montelukast sodium (98% purity, CAS No. 151767-02-1), rafoxanide (99% purity, CAS No. 22662-39-1), rezvilutamide (99% purity, CAS No. 1572045-62-5), roflumilast (99% purity, CAS No. 162401-32-3), asunaprevir (99% purity, CAS No. 630420-16-5), cariprazine (99% purity, CAS No. 839712-12-8) and dovitinib (99% purity, CAS No. 405169-16-6) were purchased from MCE (Monmouth Junction, NJ, USA).

### 4.8. Ubiquitin Rhodamine 110 Enzymatic Assay

USP7 inhibitory activity was evaluated using a fluorescence-based assay monitoring the cleavage of a monoubiquitinated substrate Ub–Rho110 in black flat-bottom 384-well plates (Greiner Bio-One GmbH, Frickenhausen, Germany, #781076). In the primary screening assay, USP7-FL (BPS Bioscience, San Diego, CA, USA, #80395; 1.25 nM) in assay buffer (50 mM Tris–HCl, pH 8.0; 50 mM NaCl; 0.002% Tween-20; 5 mM DTT) was pre-incubated for 1 h with 35 compounds selected from the virtual screening workflow at a final concentration of 10 µM. Percentage inhibition was determined from fluorescence readouts relative to controls. XL188 was used as the positive control (reference inhibitor), DMSO served as the negative control, and assay buffer without enzyme was used as the no-enzyme control. Based on the primary screening results, four compounds were selected for dose–response analysis. IC_50_ values were determined using a six-point, two-fold serial dilution (0–100 µM) following pre-incubation of USP7-FL (1.25 nM) or USP7-CD (MCE, Sollentuna, Sweden, #HY-P74478; 125 nM) with the test compounds under identical assay conditions. The reactions were pre-incubated, under light protection, during 60 min at room temperature prior to the addition of 250 nM of Ub-Rho110 (BPS Bioscience, San Diego, CA, USA, #81151) substrate solution in assay buffer, final volume of 25 μL. The initial rate of the reaction was measured by collecting fluorescence data at five-minute intervals over a 90-min period using EnSpire multimode plate reader (Perkin Elmer, Walthan, MA, USA) at excitation and emission wavelengths of 485 nm and 535 nm, respectively. After 90 min incubation at room temperature, the reactions were quenched with the addition of 12.5 µL of stop buffer (assay buffer + 0.6% trifluoroacetic acid) and fluorescence was recorded. Curve fitting and IC_50_ calculations were determined using GraphPad Prism v10.4.2 (San Diego, CA, USA). IC_50_ values were calculated from three independent biological experiments, each performed in technical triplicate.

#### Assay Interference Control Experiments

To evaluate potential assay interference, complementary control experiments were performed at the highest compound concentration used in the dose–response assays (100 μM). All fluorescence measurements were acquired using the same excitation and emission settings as those employed for the USP7 inhibition assay (485 nm and 535 nm, respectively). Intrinsic compound fluorescence was evaluated by incubating each compound in assay buffer in the absence of enzyme and substrate. Background fluorescence was measured and corrected by subtraction of the assay buffer signal. To evaluate potential effects on substrate fluorescence, compounds were also incubated with intact Ub-Rho110 in assay buffer in the absence of USP7, and fluorescence was recorded under identical acquisition conditions. Potential fluorescence quenching was evaluated by first generating fluorescent Rho110 through incubation of USP7-FL with Ub-Rho110 for 90 min. Fluorescence was recorded immediately before compound addition, after which test compounds or vehicle were added and fluorescence was measured again following incubation of 30 min. The remaining fluorescence was calculated relative to the corresponding vehicle control. To assess detergent-sensitive inhibition, the USP7 enzymatic assay was repeated using assay buffer containing either the standard Tween-20 concentration (0.002%) or a ten-fold higher concentration (0.02%). Percentage inhibition was calculated relative to the corresponding DMSO control for each detergent condition.

### 4.9. In Silico ADME and Drug-likeness Prediction

Physicochemical and ADME properties of the selected hits were predicted in silico using the SwissADME web server (Swiss Institute of Bioinformatics, Lausanne, Switzerland, available at: http://www.swissadme.ch/) (accessed on 12 December 2023) [42]. The evaluated physicochemical descriptors included molecular weight (MW, g/mol), number of hydrogen bond donors (HBD), number of hydrogen bond acceptors (HBA), number of rotatable bonds, and topological polar surface area (TPSA, Å^2^). Drug-likeness was assessed based on compliance with Lipinski’s Ro5, and structural liabilities were screened using the PAINS filter. Lipophilicity was estimated as the Consensus LogP_o/w_, and aqueous solubility was predicted using the ESOL model (Log S, log mol/L). In addition, the potential for CYP3A4 inhibition was evaluated to assess possible metabolic liabilities. Overall, these descriptors were selected to provide a comprehensive assessment of drug-likeness, pharmacokinetic properties, and potential toxicity risks.

### 4.10. Cell Lines and Tissue Culture

Human breast cancer cells MDA-MB-231, Sk-Br-3 and MCF-7, human lung cancer cells A549, H460 and H1437, and human pancreatic cancer cells PANC-1 and AsPc-1 were purchased from American Type Culture Collection (ATCC, Manassas, VA, USA). Epithelial cell line MCF10A was purchased from ATCC (Manassas, VA, USA).

MDA-MB-231, Sk-Br-3, MCF-7, MCF10A and A549 cells were cultured in DMEM (Dulbecco’s Modified Eagle’s Medium)/Hams F-12 50/50 Mix (Corning Cellgro, Manassas, VA, USA). PANC-1 cells were cultured in DMEM (Corning Cellgro, Manassas, VA, USA). H460, H1437 and AsPc-1 cells were cultured in RPMI 1640 (Corning Cellgro, Manassas, VA, USA). All cell culture formulations were supplemented with 10% fetal bovine serum (FBS, VWR; Radnor, PA, USA) and 1% penicillin and streptomycin (P/S) (Corning Cellgro, Manassas, VA, USA). Trypsin-EDTA 0.25% (Corning Cellgro, Manassas, VA, USA) was used to dissociate cells for passaging upon reaching confluence. All cell lines were maintained in a humidified incubator with 5% atmospheric CO_2_ at 37 °C and used within six to seven passages after thawing.

### 4.11. Cell Viability

MDA-MB-231 and A549 cells were plated at a density of 7500 cells per well. Sk-Br-3 and H1437 cells were plated at a density of 10,000 cells per well. MCF-7 and PANC-1 cells were plated at a density of 12,500 cells per well. H460 and AsPc-1 were plated at a density of 15,000 cells per well. MCF10A were plated at a density of 30,000 cells per well. All cells were plated in 96-well plates (Corning, NY, USA, #353072) and allowed to adhere overnight. The following day, cells were treated with candesartan cilexetil, closantel, montelukast sodium, or rafoxanide using a nine-point, two-fold serial dilution (0–200 µM). The maximum concentration tested (200 µM) was not limited by compound solubility under the assay conditions. The final DMSO concentration was maintained at 1% (*v*/*v*) in all treatment and vehicle control wells. At 72 h post-treatment, 50 μL of MTT (3-[4,5-dimethylthiazol-2-yl]-2,5-diphenyltetrazolium bromide; Sigma-Aldrich, Damstadt, Germany) was added, and the plates were incubated at 37 °C for approximately 4 h. The supernatant was then removed, and 100 μL of developing solution (0.04 N HCl in isopropanol) was added. Samples were measured in a VersaMax Microplate Reader (Molecular Devices, San Jose, CA, USA). Absorbance was measured at 570 nm, and data analysis was performed using SoftMax Pro 5.4.1 software (Molecular Devices). Dose–response curves were fitted by nonlinear regression using GraphPad Prism v10.4.2 (San Diego, CA, USA). IC_50_ values were calculated from three independent biological experiments, each performed in technical triplicate.

## 5. Conclusions

The present study identified four active compounds (>50% inhibition) and twenty-one weakly active compounds (10–49% inhibition) exhibiting biochemical USP7 inhibitory activity through an integrated workflow combining pharmacophore modelling, structure-based virtual screening, molecular docking, and biochemical validation. Four selected candidate USP7-inhibitory scaffolds demonstrated antiproliferative activity across a panel of human cancer cell lines, together with a favorable preliminary selectivity profile. Although the present study does not establish that these cellular effects are mediated directly by USP7 inhibition, the combined computational, biochemical, and cellular findings support the prioritization of these scaffolds for further mechanistic investigation and hit-to-lead optimization.

Molecular docking suggests that the validated hits occupy pharmacologically relevant region of the USP7 catalytic cleft and establish interactions with key residues involved in ligand recognition. Among the validated hits, rafoxanide demonstrated the most favorable overall profile, combining predicted USP7 binding and cellular antiproliferative activity. To the best of our knowledge, this study is the first to report candesartan cilexetil, montelukast sodium, rafoxanide, and closantel as compounds exhibiting biochemical USP7 inhibitory activity.

Although substantial optimization and orthogonal validation will be required to improve potency, selectivity, and drug-like properties and to confirm direct USP7 engagement, these findings expand the chemical diversity of candidate USP7 inhibitors and provide new starting points for medicinal chemistry efforts. Future studies will focus on elucidating the mechanisms of action of these scaffolds, establishing their selectivity across the deubiquitinase family, and optimizing their pharmacological properties to enable the development of more potent and selective USP7 inhibitors.

## Figures and Tables

**Table 1:** Pharmacophore models validation metrics using a dataset of actives and decoys. A: total number of actives in the dataset, D: total number of compounds in the dataset, d: total number of decoys in the dataset, Ht: total number of compounds retrieved by the models, TP: true positive, TN: true negative, FP: false positive, FN: false negative, Se: sensitivity, Sp: specificity, EF: enrichment factor, EF_0.5%_: enrichment factor at 0.5% of the screened database, EF_5%_: enrichment factor at 5% of the screened database, PM: power metric.

	Pharmacophore Models			
	Hyp_A1	Hyp_A2	Hyp_A5	Hyp_B4
A	26	26	26	29
D	1318	1318	1318	2077
d	1292	1292	1292	2048
Ht	27	44	27	49
TP	24	25	17	11
TN	1289	1273	1282	2010
FP	3	19	10	38
FN	2	1	9	18
% Accuracy	99.62	98.48	98.56	97.30
% Se	92.31	96.15	65.38	37.93
% Sp	99.77	98.53	99.23	98.14
EF	45.06	28.80	31.92	16.08
EF_0.5%_	53.85	53.85	53.85	68.97
EF_5%_	8.46	9.23	3.08	0.69
PM	1.00	0.98	0.99	0.95

**Table 2:** Representation of the four best pharmacophore hypotheses and their inter-feature distances.

Hypothesis	Matching Mode	Features	Inter-Features Distance (Å)
Hyp_A1	Total Match Compounds have to fulfill all four features	F1, F2, F3, F4; ExcV	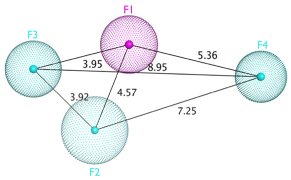
Hyp_A2	Partial Match Compounds have to fulfil at least three of the four features	F1, F2, F3, F4; ExcV Essential: F1, F2, F3; Constraint: at least three	
Hyp_A5	Partial Match Compounds have to fulfil at least four of the five features	F1, F2, F3, F5, F6, ExcV Essential: F1, F2, F3; Constraint: at least four	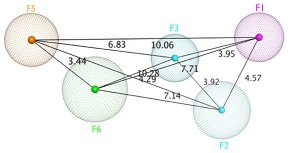
Hyp_B4	Total Match Compounds have to fulfill all five features	F1, F2, F3, F4, F6, ExcV	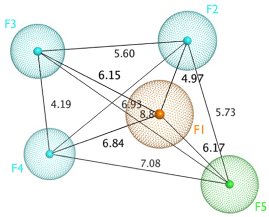

**Table 3:** Inhibitory activity of the selected in silico compounds against recombinant full-length USP7 (USP7-FL) at 10 µM. Percent inhibition values are presented as mean ± SEM from at least three independent experiments. XL188 was included as a reference inhibitor. The control corresponds to the enzymatic activity measured in the absence of inhibitor. Compounds were classified as active (>50% inhibition), weakly active (10–49% inhibition), or inactive (<10% inhibition). NA: not active.

Compound	Compound’s Source	Average Percent Inhibition at 10 µM	Activity Classification	MOE Docking Score
XL188	Sigma	96.8 ± 0.9	Reference inhibitor	−10.1
Closantel	MCE	63.2 ± 2.0	Active	−8.98
Montelukast sodium	MCE	56.8 ± 2.3	Active	−8.71
Rafoxanide	MCE	55.3 ± 3.1	Active	−8.68
Candesartan cilexetil	MCE	51.7 ± 2.5	Active	−8.50
EA6	In-house	37.7 ± 2.6	Weakly active	−7.92
EA18	In-house	37.2 ± 2.1	Weakly active	−6.84
EA17	In-house	36.4 ± 3.6	Weakly active	−7.35
EA21	In-house	35.8 ± 2.1	Weakly active	−6.82
EA26	In-house	35.1 ± 2.5	Weakly active	−6.78
EA12	In-house	35.1 ± 3.3	Weakly active	−6.67
EA33	In-house	34.3 ± 2.4	Weakly active	−5.82
EA23	In-house	33.8 ± 2.6	Weakly active	−6.32
EA16	In-house	33.8 ± 2.3	Weakly active	−6.2
EA20	In-house	33.6 ± 2.5	Weakly active	−6.3
EA34	In-house	33.6 ± 3.4	Weakly active	−6.45
EA28	In-house	32.3 ± 2.1	Weakly active	−5.98
EA2	In-house	31.7 ± 2.3	Weakly active	−5.74
EA25	In-house	31.5 ± 2.7	Weakly active	−5.85
EA35	In-house	31.3 ± 2.3	Weakly active	−6.21
EA7	In-house	31.0 ± 2.4	Weakly active	−5.87
EA13	In-house	30.6 ± 2.2	Weakly active	−6.63
EA3	In-house	30.1 ± 2.5	Weakly active	−5.68
EA29	In-house	28.2 ± 2.8	Weakly active	−5.79
EA5	In-house	27.1 ± 3.0	Weakly active	−5.48
Rezvilutamide	MCE	10.1 ± 1.9	Weakly active	−7.09
TAK-243	MCE	≤10	Inactive	−6.50
Roflumilast	MCE	≤10	Inactive	−5.71
Asunaprevir	MCE	≤10	Inactive	−5.60
Cariprazine	MCE	≤10	Inactive	−6.25
EA9	NCI	≤10	Inactive	−7.59
Dovitinib	MCE	≤10	Inactive	−5.85
EA19	NCI	≤10	Inactive	−7.52
EA11	NCI	NA	Inactive	−7.13
EA31	NCI	NA	Inactive	−6.8
EA15	NCI	NA	Inactive	−7.37

**Table 4:** Inhibitory activity of the four candidate USP7-inhibitory scaffolds against recombinant full-length USP7 (USP7-FL) and the USP7 catalytic domain (USP7-CD). The IC_50_ values are reported as mean ± SEM from at least three independent experiments. Control corresponds to enzymatic activity measured in the absence of inhibitor. FL: full-length; CD: catalytic domain.

Compounds	IC_50_ USP7 FL (μM)	IC_50_ USP7 CD (μM)
Candesartan cilexetil	32.89 ± 2.91	20.35 ± 1.81
Montelukast sodium	29.00 ± 1.89	15.71 ± 2.13
Rafoxanide	25.00 ± 0.21	23.44 ± 1.88
Closantel	18.15 ± 1.68	10.08 ± 1.03

**Table 5:** Type of interactions of the four candidate USP7-inhibitory scaffolds within the USP7 binding site. Residues Gln297, Arg301, Asn418, Lys420, Asp459, Asn460, His461, and Phe409 are involved in the ligand-protein interactions.

Compounds	Type of Interaction	Energy of Interaction (Kcal/mol)
Candesartan cilexetil	Arg301—electrostatic interactions	−1.5; −2.0; −2.6; −3.1; −3.7; −4.5
	Gln297—hydrogen bond interaction	−0.8
Montelukast sodium	Arg301—electrostatic interactions	−0.8; −1.0; −1.8
	Gln297—hydrogen bond interaction	−5.8
	Met407—π··· C–H interaction	−1.1
	Phe409—π··· C–H interaction	−0.5
Rafoxanide	Gln297—halogen bond interaction	−0.7
	Asp459—hydrogen bond interaction	−1.9
	His461—π··· C–H interaction	−0.5
Closantel	Gln297—halogen bond interaction	−1.8
	Lys420—hydrogen bond interaction	−1.2
	His461—hydrogen bond interaction	−2.7
	Asn460—hydrogen bond interaction	−0.5
	Asn418—hydrogen bond interaction	−4.2

**Table 6:** SwissADME-predicted physicochemical and pharmacokinetic properties of the candidate USP7-inhibitory compounds identified.

Parameter	Candesartan Cilexetil	Montelukast Sodium	Closantel	Rafoxanide
Molecular weight (MW)	608.64 g/mol	584.17 g/mol	661.06 g/mol	625.00 g/mol
Hydrogen bond donor (HBD)	0	1	1	1
Hydrogen bond acceptor (HBA)	11	4	3	3
Rotatable bonds	13	12	5	5
TPSA	140.44 Å^2^	98.55 Å^2^	75.95 Å^2^	61.39 Å^2^
Lipinski rule	No (3 violations)	No (2 violations)	No (2 violations)	No (2 violations)
PAINS	0	0	0	0
Consensus LogP_o/w_	4.46	5.92	5.50	6.02
Log S (ESOL)	−7.66	−7.93	−8.60	−8.31
CYP3A4 inhibitor	Yes	Yes	No	No

**Table 7:** Antiproliferative activity of the validated hit compounds determined by the MTT cell viability assay. Growth-inhibitory activity was evaluated in human breast (MDA-MB-231, Sk-Br-3, and MCF-7), lung (A549, H460, and H1437), and pancreatic (PANC-1 and AsPc-1) cancer cell lines. The non-tumorigenic human mammary epithelial cell line MCF10A was included to provide a preliminary assessment of selectivity. IC_50_ values are presented as mean ± SEM from three independent biological experiments, each performed in technical triplicate.

Cancer Type	Cell Line	Candesartan Cilexetil IC_50_ (μM)	Montelukast Sodium IC_50_ (μM)	Closantel IC_50_ (μM)	Rafoxanide IC_50_ (μM)
Breast cancer	MDA-MB-231	52.27 ± 1.02	52.87 ± 0.68	25.80 ± 0.55	25.60 ± 0.30
	Sk-Br-3	49.09 ± 0.36	52.91 ± 0.59	51.37 ± 1.20	33.12 ± 1.60
	MCF-7	51.17 ± 0.78	86.73 ± 5.30	48.59 ± 2.36	21.27 ± 2.31
Lung cancer	A549	89.28 ± 9.38	66.22 ± 6.03	50.70 ± 0.51	24.46 ± 0.48
	H460	57.60 ± 3.55	49.26 ± 0.53	43.61 ± 1.78	30.72 ± 1.04
	H1437	21.19 ± 0.97	52.96 ± 0.16	36.72 ± 0.75	11.24 ± 0.01
Pancreatic cancer	PANC-1	90.11 ± 3.80	72.37 ± 3.38	41.06 ± 6.76	22.78 ± 0.77
	AsPc-1	50.17 ± 0.67	47.17 ± 0.34	19.91 ± 3.32	24.31 ± 0.04
Non-tumoral	MCF10A	92.87 ± 1.20	73.78 ± 1.85	82.78 ± 1.80	131.56 ± 1.35

**Table 8:** Selectivity index (SI) of the validated candidate USP7-inhibitory scaffolds. SI was calculated as the ratio of the IC_50_ value in the non-tumorigenic human mammary epithelial cell line MCF10A to the IC_50_ value in each cancer cell line. Higher SI values indicate greater preferential antiproliferative activity toward cancer cells.

Cancer Type	Cell Line	Candesartan Cilexetil	Montelukast Sodium	Closantel	Rafoxanide
Breast cancer	MDA-MB-231	1.78	1.40	3.21	5.14
	Sk-Br-3	1.89	1.39	1.61	3.97
	MCF-7	1.81	0.85	1.70	6.19
Lung cancer	A549	1.04	1.11	1.63	5.38
	H460	1.61	1.50	1.90	4.28
	H1437	4.38	1.39	2.25	11.70
Pancreatic cancer	PANC-1	1.03	1.02	2.02	5.78
	AsPc-1	1.85	1.56	4.16	5.41

**Table 9:** Summary of the key biological and physicochemical properties of the validated candidate USP7-inhibitory scaffolds. The table summarizes the biochemical activity against recombinant USP7, antiproliferative activity, preliminary selectivity, predicted physicochemical properties, and principal optimization priorities for the four validated candidate USP7-inhibitory scaffolds. SI: selectivity index.

Compound	USP7-FL IC_50_ (µM)	Best Cellular IC_50_ (µM) (Cell Line)	SI	Lipinski Violations	Consensus LogP	Predicted Solubility (LogS)	CYP3A4 Inhibitor	Key Optimization Priorities
Candesartan cilexetil	32.89 ± 2.91	21.19 ± 0.97 (H1437)	4.38	3	4.46	−7.66	Yes	Improve potency and physicochemical properties.
Montelukast sodium	29.00 ± 1.89	47.17 ± 0.34 (AsPc-1)	1.56	2	5.92	−7.93	Yes	Improve potency; reduce lipophilicity and improve aqueous solubility.
Closantel	18.15 ± 1.68	19.91 ± 3.32 (AsPc-1)	4.16	2	5.50	−8.60	No	Improve aqueous solubility and potency.
Rafoxanide	25.00 ± 0.21	11.24 ± 0.01 (H1437)	11.70	2	6.02	−8.31	No	Reduce lipophilicity and improve aqueous solubility.

## Data Availability

The original contributions presented in this study are included in the article/Supplementary Material. Further inquiries can be directed to the corresponding author.

## Supplementary Materials

The following supporting information can be downloaded at: https://www.mdpi.com/article/10.3390/ph19081219/s1, Figure S1: Superposition of FTMap probe clusters within the substrate-binding pocket of USP7 crystal structures. The co-crystallized inhibitors ALM4 (5N9T), L55 (6M1K), Compound **18** (6VN2), and Compound **14** (6VN6) are shown as yellow stick representations. The protein structure is omitted for clarity; Figure S2: Representation of the main (A) and secondary (B) hot spots within the substrate-binding site of USP7 crystal structures. Each consensus site (CS) is depicted as a collection of probes corresponding to the centers of the clustered probe molecules. Probe clusters are colored according to rank, from highest to lowest: light blue, dark blue, red, dark green, brown, light green, turquoise, and magenta. The superposed USP7 structures are shown as grey ribbon representations.; Figure S3: Validation of the docking protocol by self-docking. Superposition of the best-docked pose of ligand L55 (orange) with its crystallographic conformation (cyan), resulting in an RMSD of 0.68 Å. Docking was performed in MOE 2022.02 using the following docking parameters: Placement: Triangle Matcher, Scoring function: London dG; Refinement: Rigid Receptor, Scoring function: GBVI/WSA dG; Figure S4: Dose–response curves of the four selected candidate USP7 inhibitors. Purple curves correspond to the full-length USP7 (USP7-FL) construct, whereas blue curves correspond to the catalytic domain construct (USP7-CD); Figure S5: Assessment of intrinsic compound fluorescence. (A) Background-corrected fluorescence of compounds (100 μM) in assay buffer under the excitation and emission settings used for the USP7 assay. (B) Background-corrected fluorescence of intact Ub-Rho110 in the presence of each compound (100 μM). Data represent mean ± SEM from three independent experiments, each performed in technical triplicate; Figure S6: Assessment of fluorescence quenching by candidate USP7-inhibitory scaffolds. Fluorescent Rh110 generated by USP7-mediated cleavage of Ub-Rho110 was incubated with each compound (100 μM), and fluorescence was measured immediately before and 30 min after compound addition. Fluorescence values were normalized to the corresponding DMSO control. Data represent mean ± SEM from three independent experiments, each performed in technical triplicate; Figure S7: Assessment of detergent sensitivity of candidate USP7-inhibitory scaffolds. USP7 inhibitory activity was evaluated at 100 μM in assay buffer containing either the standard Tween-20 concentration (0.002%) or an increased Tween-20 concentration (0.02%). Percent inhibition was calculated relative to the corresponding DMSO control for each detergent condition. Data represent mean ± SEM from three independent experiments, each performed in technical triplicate; Table S1: 2D ligand-receptor interaction diagrams of the four PDB complexes used in the hotspot prediction studies. Ligand–protein interactions are depicted as follows: green arrows indicate side-chain hydrogen bond donor/acceptor interactions, while blue arrows represent backbone hydrogen bond donor/acceptor interactions. Green dashed lines with an arene symbol denote C–H···π interactions. Binding site residues are color-coded according to their physicochemical properties: hydrophobic residues are shown in green, polar residues in purple, while blue and red contours indicate basic and acidic residues, respectively. The 2D interaction diagrams were generated using MOE 2022.02 (Chemical Computing Group); Table S2: Distribution of FTMap probe clusters within the substrate-binding site of USP7 crystal structures. The co-crystallized inhibitors ALM4, L55, Compound 18, and Compound 14 are shown as yellow stick representations. The protein structure is omitted for clarity; Table S3: 2D ligand-receptor interactions diagram of the PDB complexes used in pharmacophore model A and B hypotheses. Ligand–protein interactions are depicted as follows: green arrows indicate side-chain hydrogen bond donor/acceptor interactions, while blue arrows represent backbone hydrogen bond donor/acceptor interactions. Green dashed lines with an arene symbol denote C–H···π interactions. Binding site residues are color-coded according to their physicochemical properties: hydrophobic residues are shown in green, polar residues in purple, while blue and red contours indicate basic and acidic residues, respectively. The 2D interaction diagrams were generated using MOE 2022.02 (Chemical Computing Group); Table S4: MOE scoring and USP7 percent inhibition data from the thirty-five compounds tested in vitro. NA: not active; Table S5: Nonlinear regression parameters for dose–response analyses of the four validated compounds against USP7-FL and USP7-CD. NE: confidence limit not estimable by nonlinear regression because the profile likelihood did not converge within the tested parameter range.

## Abbreviations

The following abbreviations are used in this manuscript:

Acc	Hydrogen bond acceptor
Aro|PiR	Aromatic center or π ring normal
bRo5	Beyond Lipinski’s rule of 5
Cat&Don	Cation and Hydrogen bond donor
CSs	Consensus sites
D	Total number of compounds in the dataset
d	Total number of decoys in the dataset
Don&Acc	Hydrogen bond donor and acceptor
DUBs	Deubiquitinating enzymes
DUD-E	Database of Useful Decoys-Enhanced
E1	Ubiquitin-activating
E2	Ubiquitin-conjugating
E3	Ubiquitin ligase
EF	Enrichment factor
EF_0.5%_	Enrichment factor at 0.5% of the screened database
EF_5%_	Enrichment factor at 5% of the screened database
ESOL	Log S
ExcV	Excluded volumes
FN	False negative
FP	False positive
FPR	False positive rate
HAUSP	Herpesvirus-associated ubiquitin specific protease
HBA	Hydrogen bond acceptor
HBD	Hydrogen bond donor
Ht	Total number of compounds retrieved by the models
MCE	MedChemExpress
mdb	MOE molecular database
MOE	Molecule operating environment
NMR	Nuclear Magnetic Resonance
PDB	Protein Data Bank
PM	Power metric
RCSB	Research Collaboratory for Structural Bioinformatics
RMSD	Root mean square deviation
Ro5	Lipinski’s rule of 5
Se	Sensitivity
Sp	Specificity
Sp1	Subpocket 1
Sp2	Subpocket 2
Sp3	Subpocket 3
TN	True negative
TP	True positive
TPR	True positive rate
Ub-Rho110	Ubiquitin-rhodamine 110
Ubal	Ubiquitin-aldehyde
UBL	Ubiquitin-like
UPS	Ubiquitin–proteasome system
USP7	Ubiquitin-specific protease 7
USP-CD	USP7 catalytic domain
USP7-FL	USP7 full-length
