# Supplementary material for: Virtual Screening–Guided Identification of Candidate USP7-Inhibitory Scaffolds Exhibiting Antiproliferative Activity
Source: Pharmaceuticals (Basel). 2026 Aug 3;19(8):1219. doi: 10.3390/ph19081219 (PMC13516235; doi:10.3390/ph19081219)
Supplement: Supplementary file 1 [file pharmaceuticals-19-01219-s001.zip › Supplementary material_tables.pdf]

Table S1. 2D ligand-receptor interaction diagrams of the four PDB complexes used in the hotspot prediction studies. Ligand–protein interactions are depicted as follows: green arrows indicate side-chain hydrogen bond donor/acceptor interactions, while blue arrows represent backbone hydrogen bond donor/acceptor interactions. Green dashed lines with an arene symbol denote C–H···π interactions. Binding site residues are color-coded according to their physicochemical properties: hydrophobic residues are shown in green, polar residues in purple, while blue and red contours indicate basic and acidic residues, respectively. The 2D interaction diagrams were generated using MOE 2022.02 (Chemical Computing Group).

PDB ID	Ligand	Resolution	Type of interactions	2D Receptor-Ligand interactions
5N9T<br>(2017)	ALM4	1.73 Å<br>(X-Ray)	•Asp295 (sidechain H-acceptor)(O··OH)(2.75 Å)(-3.8 kcal/mol)<br>•Val296 (backbone H-donor)(NH··O)(3.07 Å)(-2.2 kcal/mol)<br>•Gln297 (sidechain H-donor)(NH··N)(3.06 Å)(-3.2 kcal/mol)<br>•Gln351 (sidechain H-acceptor)(O··NH)(2.84 Å)(-10.9 kcal/mol)<br>•Gln351 (C-H··· π interaction)(3.69 Å)(-0.6 kcal/mol)<br>•Met407 (C-H··· π interaction)(4.07 Å)(-0.8 kcal/mol)<br>•Arg408 (backbone H-donor)(NH··O)(3.13 Å)(-0.8 kcal/mol)<br>•Phe409 (backbone H-donor)(NH··O)(2.95 Å)(-3.8 kcal/mol)<br>•Phe409 (backbone H-donor)(NH··N)(3.31 Å)(-0.6 kcal/mol)<br>•Tyr465 (sidechain H-donor)(OH··O)(2.67 Å)(-3.1 kcal/mol)	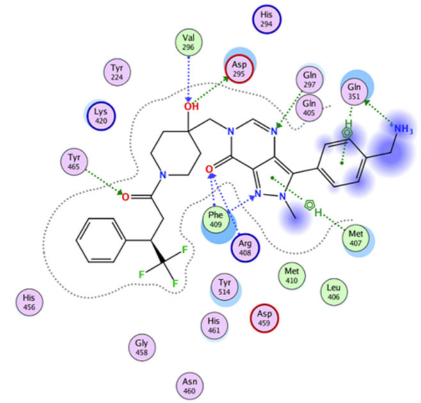
6M1K<br>(2021)	L55	2.25 Å<br>(X-Ray)	•Asp295 (sidechain H-acceptor)(O··OH)(2.73 Å)(-4.0 kcal/mol)<br>•Val296 (backbone H-donor)(NH··O) (3.01 Å)(-2.4 kcal/mol)<br>•Gln297 (sidechain H-donor)(NH··N)(3.05 Å)(-2.9 kcal/mol)<br>•Gln351 (sidechain H-acceptor)(O··NH)(2.84 Å)(-11.9 kcal/mol)<br>•Gln351 (C-H··· π interaction)(3.66 Å)(-1.0 kcal/mol)<br>•Phe409 (backbone H-donor)(NH··N)(3.64 Å)(-0.8 kcal/mol)<br>•Met407 (C-H··· π interaction)(9.89 Å)(-1.3 kcal/mol)	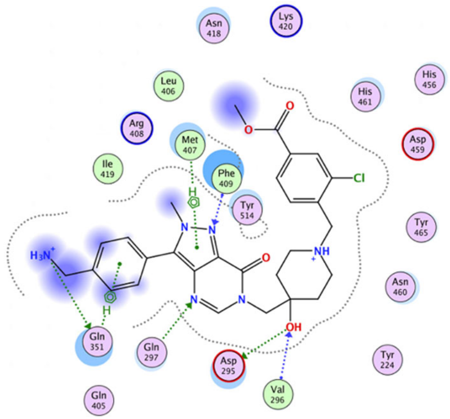

PDB ID	Ligand	Resolution	Type of interactions	2D Receptor-Ligand interactions
6VN2 (2020)	Compound 18	2.93 Å (X-Ray)	•Asp295 (sidechain H-acceptor)(O··NH)(2.77 Å)(-9.1 kcal/mol)<br>•Asp295 (salt bridge)(O··NH) (2.77 Å)(-6.2 kcal/mol)<br>•Val296 (backbone H-donor)(NH··N) (3.05 Å)(-4.3 kcal/mol)<br>•Gln297 (sidechain H-donor)(NH··O) (2.87 Å)(-4.5 kcal/mol)<br>•Phe409 (backbone H-donor)(NH··O) (2.89 Å)(-3.5 kcal/mol)<br>•His456 (π··· C-H interaction) (3.87 Å)(-0.9 kcal/mol)<br>•Tyr514 (C-H··· π interaction) (4.40 Å)(-1.1 kcal/mol)	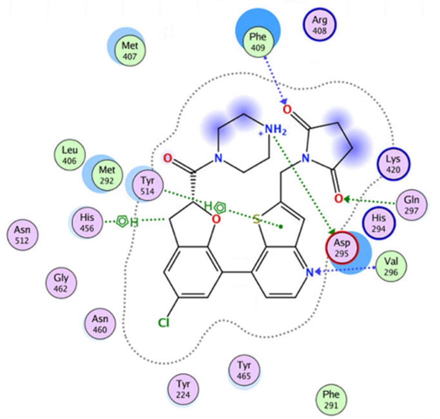
6VN6 (2021)	Compound 14	2.99 Å (X-Ray)	•Asp295 (sidechain H-acceptor)(O··NH) (2.76 Å)(-11.7 kcal/mol)<br>•Asp295 (salt bridge)(O··NH) (2.76 Å)(-6.3 kcal/mol)<br>•Val296 (backbone H-donor)(NH··N) (2.98 Å)(-5.1 kcal/mol)<br>•Gln297 (sidechain H-donor)(NH··O)(2.99 Å)(-2.3 kcal/mol)<br>•Leu406 (backbone H-acceptor)(O··OH) (2.76 Å)(-2.2 kcal/mol)<br>•Phe409 (C-H··· π interaction) (3.59 Å)(-0.5 kcal/mol)<br>•His456 (π··· C-H interaction) (4.00 Å)(-0.7 kcal/mol)<br>•Tyr514 (C-H··· π interaction) (4.65 Å)(-0.9 kcal/mol)	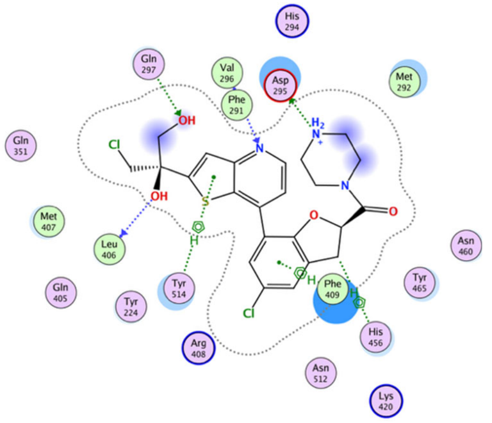

Table S2. Distribution of FTMap probe clusters within the substrate-binding site of USP7 crystal structures. The co-crystallized inhibitors ALM4, L55, Compound 18, and Compound 14 are shown as yellow stick representations. The protein structure is omitted for clarity.

PDB ID	Consensus sites (CSs)	No. of probe clusters	Structural representation
**5N9T**	CS000 (light blue)	**26**	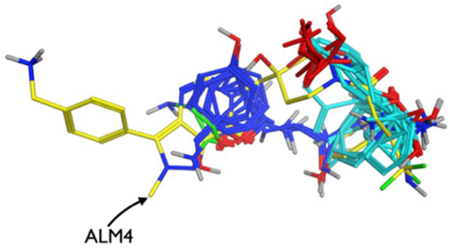
	CS001 (dark blue)	**17**	
	CS003 (red)	9	
	CS007 (light green)	3	
**6M1K**	CS000 (light blue)	**19**	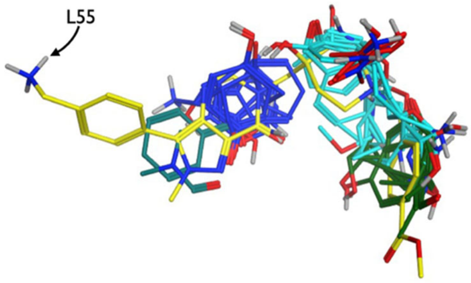
	CS001 (dark blue)	**16**	
	CS003 (dark green)	12	
	CS005 (red)	5	
	CS009 (turquoise)	2	
**6VN2**	CS000 (light blue)	**29**	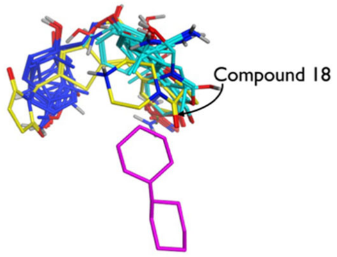
	CS001 (dark blue)	**18**	
	CS004 (red)	7	
	CS010 (magenta)	2	
**6VN6**	CS000 (dark blue)	**24**	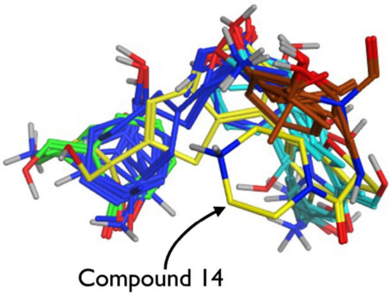
	CS001 (light blue)	11	
	CS004 (brown)	10	
	CS006 (light green)	6	
	CS007 (turquoise)	6	

Table S3. 2D ligand-receptor interactions diagram of the PDB complexes used in pharmacophore model A and B hypotheses. Ligand–protein interactions are depicted as follows: green arrows indicate side-chain hydrogen bond donor/acceptor interactions, while blue arrows represent backbone hydrogen bond donor/acceptor interactions. Green dashed lines with an arene symbol denote C–H···π interactions. Binding site residues are color-coded according to their physicochemical properties: hydrophobic residues are shown in green, polar residues in purple, while blue and red contours indicate basic and acidic residues, respectively. The 2D interaction diagrams were generated using MOE 2022.02 (Chemical Computing Group).

	**PDB ID**	**Ligand**	**Resolution**	**Type of interactions**	**2D Receptor-Ligand interactions**
**Pharmacophore Model A**	5N9T (2017)	ALM4	1.73 Å (X-Ray)	•Asp295 (sidechain H-acceptor)(O··OH)(2.75 Å)(-3.8 kcal/mol)<br>•Val296 (backbone H-donor)(NH··O)(3.07 Å)(-2.2 kcal/mol)<br>•Gln297 (sidechain H-donor)(NH··N)(3.06 Å)(-3.2 kcal/mol)<br>•Gln351 (sidechain H-acceptor)(O··NH)(2.84 Å)(-10.9 kcal/mol)<br>•Gln351 (C-H··· π interaction)(3.69 Å)(-0.6 kcal/mol)<br>•Met407 (C-H··· π interaction)(4.07 Å)(-0.8 kcal/mol)<br>•Arg408 (backbone H-donor)(NH··O)(3.13 Å)(-0.8 kcal/mol)<br>•Phe409 (backbone H-donor)(NH··O)(2.95 Å)(-3.8 kcal/mol)<br>•Phe409 (backbone H-donor)(NH··N)(3.31 Å)(-0.6 kcal/mol)<br>•Tyr465 (sidechain H-donor)(OH··O)(2.67 Å)(-3.1 kcal/mol)	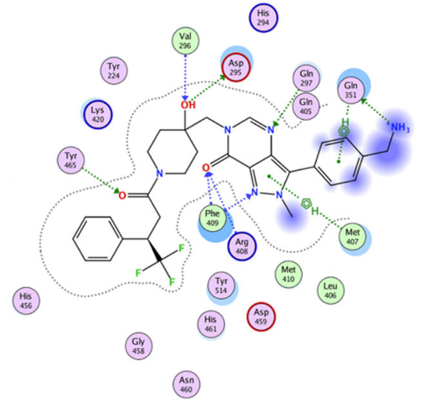
	5NGE (2017)	FT671	2.35 Å (X-Ray)	•Asp295 (sidechain H-acceptor)(O··OH)(2.74 Å)(-3.8 kcal/mol)<br>•Val296 (backbone H-donor)(NH··O)(3.12 Å)(-1.9 kcal/mol)<br>•Gln297 (sidechain H-donor)(NH··N)(3.03 Å)(-2.4 kcal/mol)<br>•Met407 (hydrophobic/sulfur-mediated contact (3.98 Å)(-0.5 kcal/mol)<br>•Met407 (C-H··· π interaction)(3.73 Å)(-1.4 kcal/mol)<br>•Phe409 (backbone H-donor)(NH··O)(2.96 Å)(-4.7 kcal/mol)<br>•Phe409 (π··· C-H interaction)(3.95 Å)(-0.5 kcal/mol)<br>•Tyr465 (sidechain H-donor)(OH··O)(2.68 Å)(-3.2 kcal/mol)<br>•Gln351 (C-H··· π interaction)(3.73 Å)(-0.5 kcal/mol)	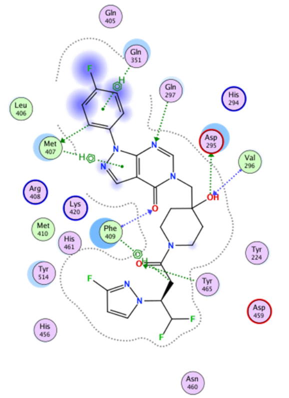

	PDB ID	Ligand	Resolution	Type of interactions	2D Receptor-Ligand interactions
**Pharmacophore Model A**	5VS6<br>(2017)	XL188	2.27 Å<br>(X-Ray)	•Asp295 (sidechain H-acceptor)(O··OH)(2.75 Å)(-3.8 kcal/mol)<br>•Val296 (backbone H-donor)(NH··O) (3.16 Å)(-1.7 kcal/mol)<br>•Gln297 (sidechain H-donor)(NH··N)(3.01 Å)(-1.9 kcal/mol)<br>•Phe409 (backbone H-donor)(NH··O)(2.90 Å)(-4.7 kcal/mol)<br>•Phe409 (π··· C-H interaction)(4.64 Å)(-0.6 kcal/mol)<br>•Tyr465 (sidechain H-donor)(OH··O)(2.66 Å)(-3.0 kcal/mol)<br>•His461 (π··· C-H interaction)(3.57 Å)(-0.7 kcal/mol)	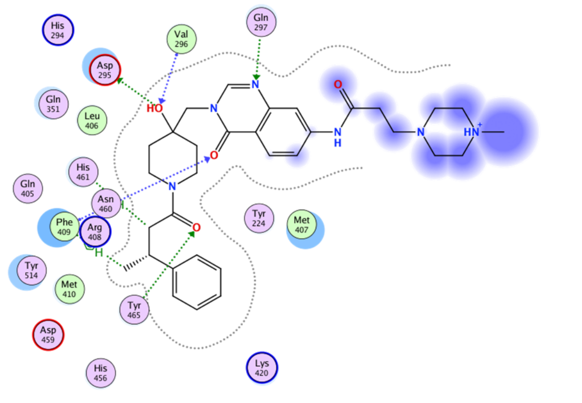
	6M1K<br>(2021)	L55	2.25 Å<br>(X-Ray)	•Asp295 (sidechain H-acceptor)(O··OH)(2.73 Å)(-4.0 kcal/mol)<br>•Val296 (backbone H-donor)(NH··O) (3.01 Å)(-2.4 kcal/mol)<br>•Gln297 (sidechain H-donor)(NH··N)(3.05 Å)(-2.9 kcal/mol)<br>•Gln351 (sidechain H-acceptor)(O··NH)(2.84 Å)(-11.9 kcal/mol)<br>•Gln351 (C-H··· π interaction)(3.66 Å)(-1.0 kcal/mol)<br>•Phe409 (backbone H-donor)(NH··N)(3.64 Å)(-0.8 kcal/mol)<br>•Met407 (C-H··· π interaction)(9.89 Å)(-1.3 kcal/mol)	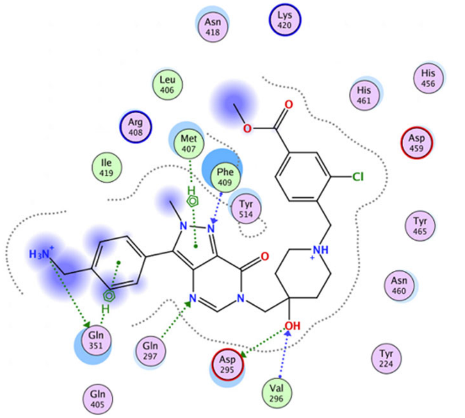

	PDB ID	Ligand	Resolution	Type of interactions	2D Receptor-Ligand interactions
**Pharmacophore Model B**	6VN2<br>(2020)	Compound 18	2.93 Å<br>(X-Ray)	•Asp295 (sidechain H-acceptor)(O··NH)(2.77 Å)(-9.1 kcal/mol)<br>•Asp295 (salt bridge)(O··NH) (2.77 Å)(-6.2 kcal/mol)<br>•Val296 (backbone H-donor)(NH··N) (3.05 Å)(-4.3 kcal/mol)<br>•Gln297 (sidechain H-donor)(NH··O) (2.87 Å)(-4.5 kcal/mol)<br>•Phe409 (backbone H-donor)(NH··O) (2.89 Å)(-3.5 kcal/mol)<br>•His456 (π··· C-H interaction) (3.87 Å)(-0.9 kcal/mol)<br>•Tyr514 (C-H··· π interaction) (4.40 Å)(-1.1 kcal/mol)	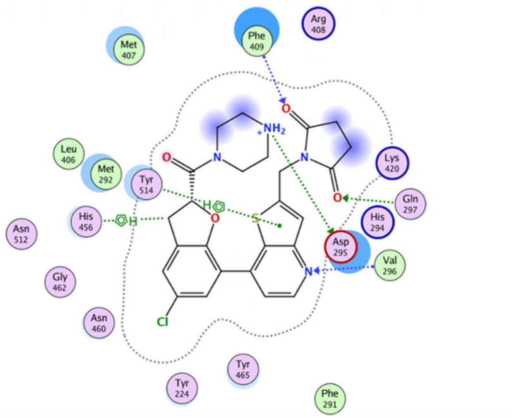
	6VN3<br>(2020)	Compound 23	2.73 Å<br>(X-Ray)	•Met292 (backbone H-acceptor)(O··CH)(3.22 Å)(-0.6 kcal/mol)<br>•His294 (sidechain H-acceptor)(O··NH) (2.78 Å)(-5.5 kcal/mol)<br>•Asp295 (sidechain H-acceptor)(O··NH)(2.79 Å)(-9.0 kcal/mol)<br>•Asp295 (salt bridge)(O··NH) (2.79 Å)(-6.1 kcal/mol)<br>•Val296 (backbone H-donor)(NH··N) (3.09 Å)(-3.8 kcal/mol)<br>•Gln297 (sidechain H-donor)(NH··O) (2.87 Å)(-4.5 kcal/mol)<br>•Phe409 (backbone H-donor)(NH··O) (2.89 Å)(-3.4 kcal/mol)<br>•Phe409 (C-H··· π interaction)(3.58 Å)(-0.5 kcal/mol)<br>•Asn512 (halogen contact)(NH··Cl)(3.46 Å)(-0.5 kcal/mol)<br>•Tyr514 (C-H··· π interaction)(4.43 Å)(-1.1 kcal/mol)	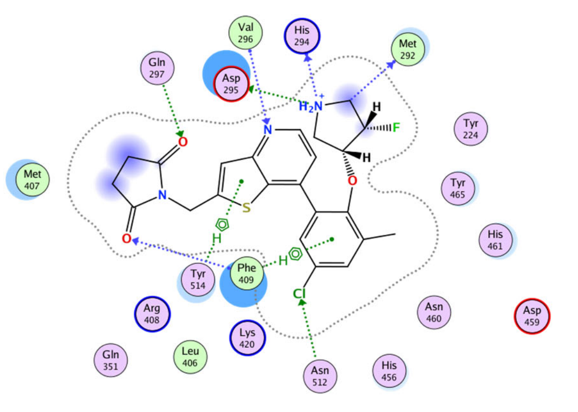

	PDB ID	Ligand	Resolution	Type of interactions	2D Receptor-Ligand interactions
**Pharmacophore Model B**	6VN5 (2020)	Compound 7	2.90 Å (X-Ray)	•His294 (sidechain H-acceptor)(O··CH) (3.10 Å)(-0.5 kcal/mol)<br>•Asp295 (sidechain H-acceptor)(O··NH)(2.77 Å)(-17.9 kcal/mol)<br>•Asp295 (salt bridge)(O··NH) (2.77 Å)(-6.2 kcal/mol)<br>•Val296 (backbone H-donor)(NH··N) (3.16 Å)(-4.1 kcal/mol)<br>•His456 (π··· C-H interaction) (3.79 Å)(-1.0 kcal/mol)	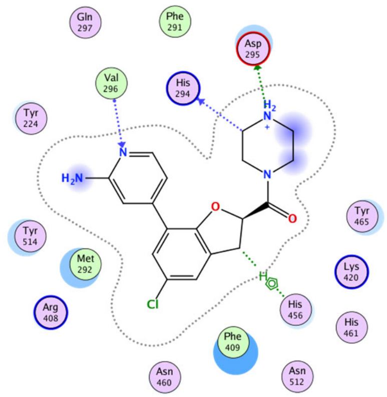
	6VN6 (2021)	Compound 14	2.99 Å (X-Ray)	•Asp295 (sidechain H-acceptor)(O··NH) (2.76 Å)(-11.7 kcal/mol)<br>•Asp295 (salt bridge)(O··NH) (2.76 Å)(-6.3 kcal/mol)<br>•Val296 (backbone H-donor)(NH··N) (2.98 Å)(-5.1 kcal/mol)<br>•Gln297 (sidechain H-donor)(NH··O)(2.99 Å)(-2.3 kcal/mol)<br>•Leu406 (backbone H-acceptor)(O··OH) (2.76 Å)(-2.2 kcal/mol)<br>•Phe409 (C-H··· π interaction) (3.59 Å)(-0.5 kcal/mol)<br>•His456 (π··· C-H interaction) (4.00 Å)(-0.7 kcal/mol)<br>•Tyr514 (C-H··· π interaction) (4.65 Å)(-0.9 kcal/mol)	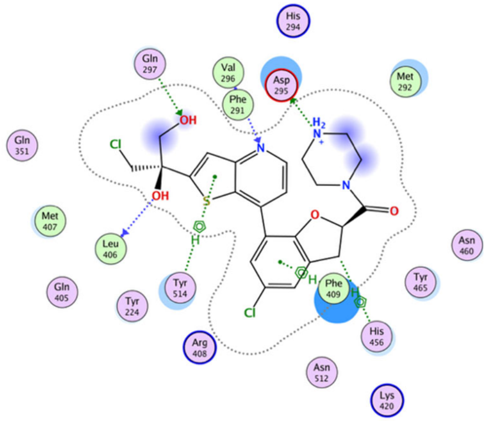

Table S4. MOE scoring and USP7 percent inhibition data from the thirty-five compounds tested *in vitro*. NA: not active.

Compound code	Compound scaffold	Average Percent Inhibition at 10 μM	MOE Docking score
Closantel	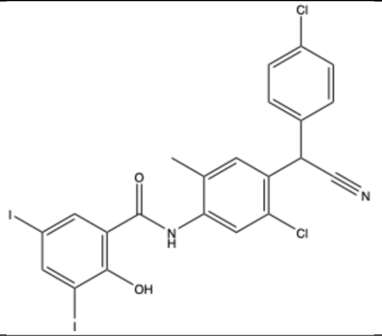	63.2 ± 2.0	-8.98
Montelukast sodium	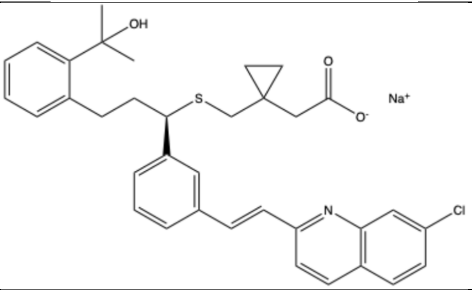	56.8 ± 2.3	-8.71
Rafoxanide	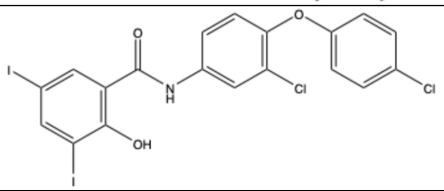	55.3 ± 3.1	-8.68
Candesartan cilexetil	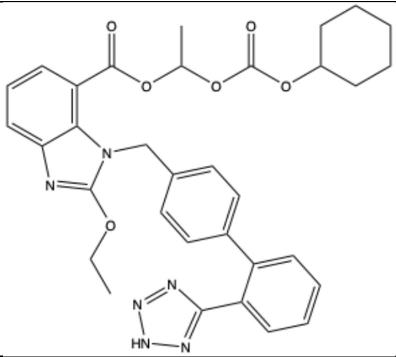	51.7 ± 2.5	-8.50
EA6	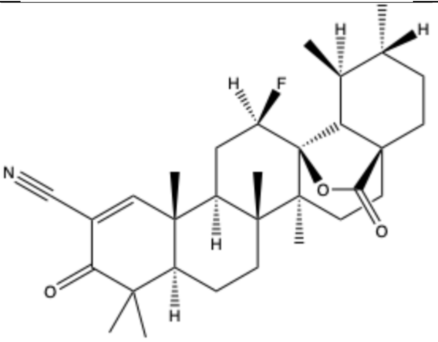	37.7 ± 2.6	-7.92

EA18	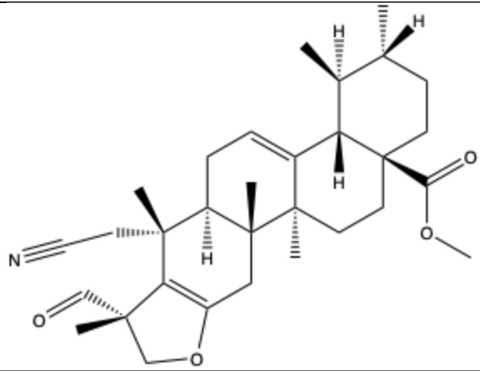	37.2 ± 2.1	-6.84
EA17	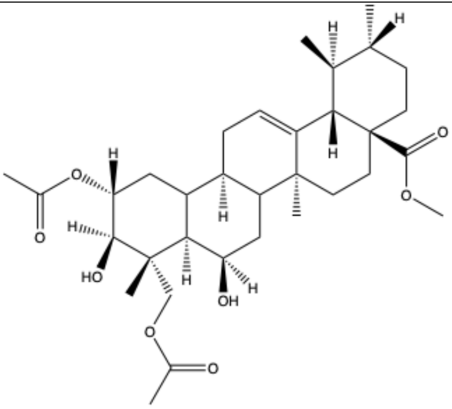	36.4 ± 3.6	-7.35
EA21	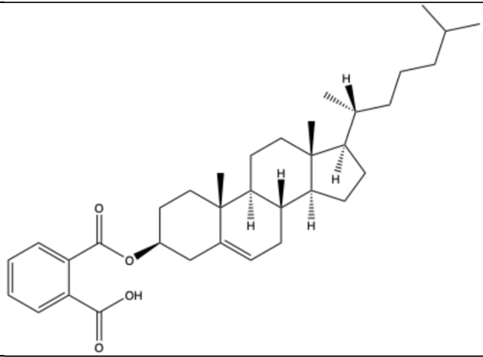	35.8 ± 2.1	-6.82
EA26	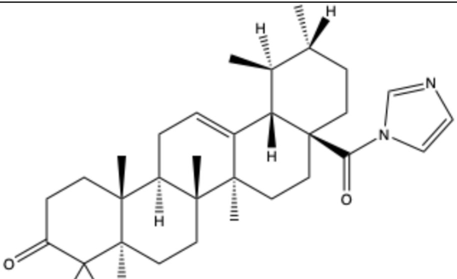	35.1 ± 2.5	-6.78
EA12	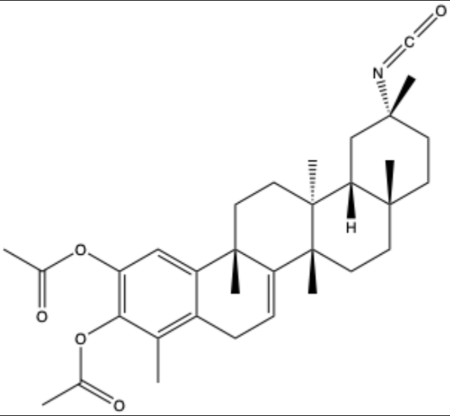	35.1 ± 3.3	-6.67

EA33	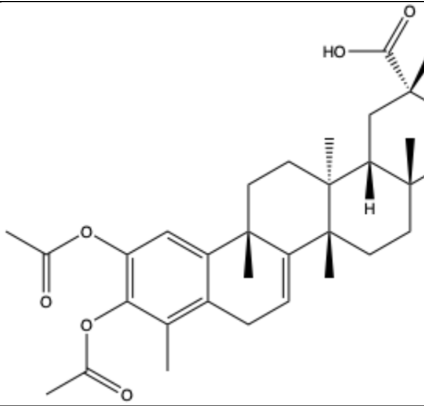	34.3 ± 2.4	-5.82
EA23	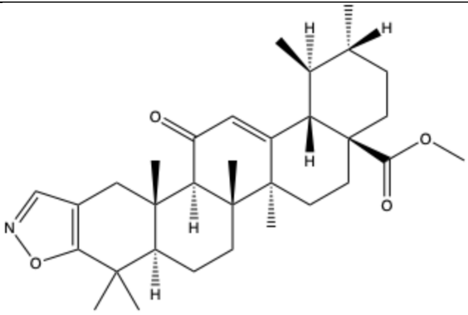	33.8 ± 2.6	-6.32
EA16	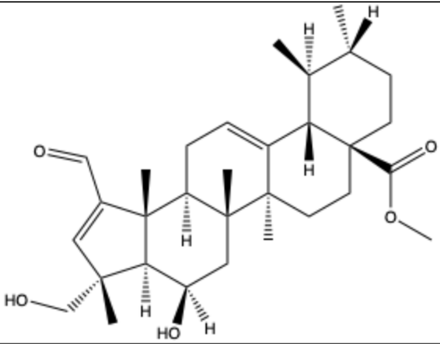	33.8 ± 2.3	-6.2
EA20	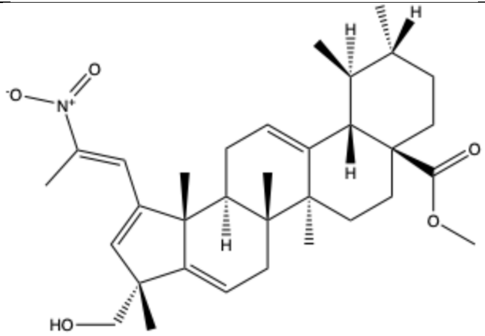	33.6 ± 2.5	-6.3
EA34	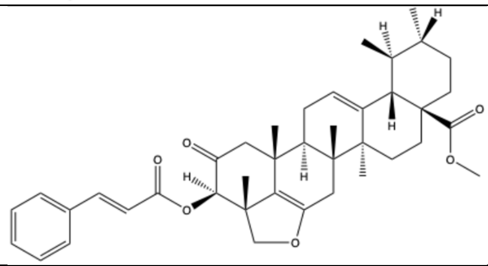	33.6 ± 3.4	-6.45

EA28	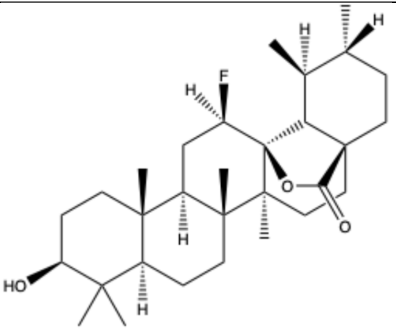	32.3 ± 2.1	-5.98
EA2	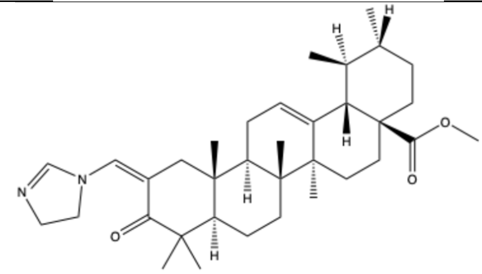	31.7 ± 2.3	-5.74
EA25	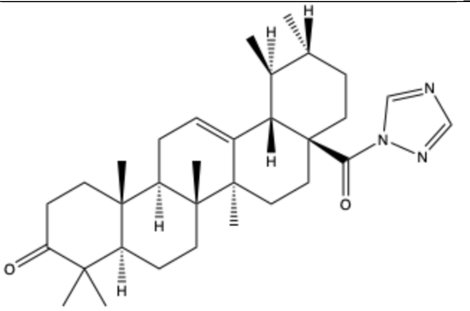	31.5 ± 2.7	-5.85
EA35	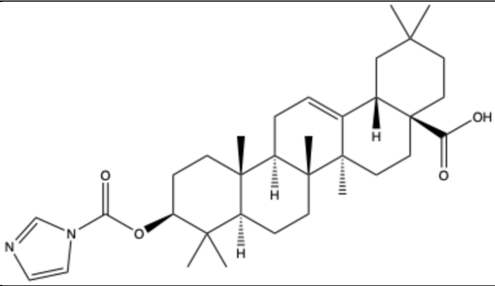	31.3 ± 2.3	-6.21
EA7	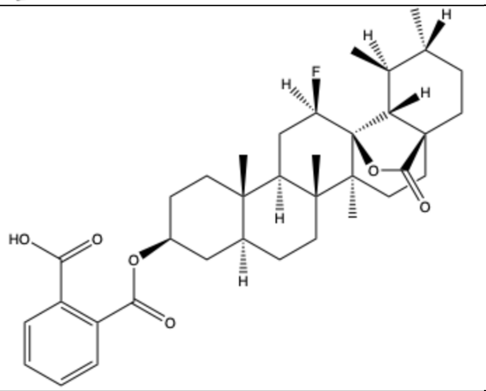	31.0 ± 2.4	-5.87

EA13	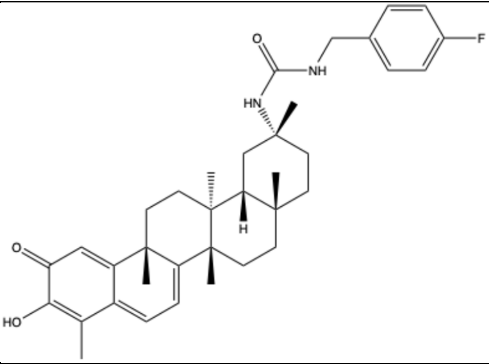	30.6 ± 2.2	-6.63
EA3	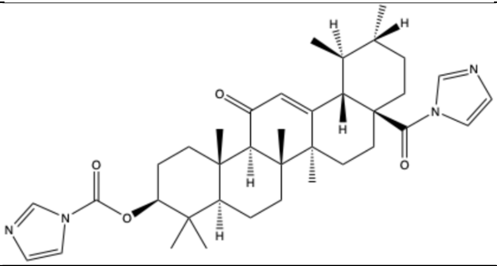	30.1 ± 2.5	-5.68
EA29	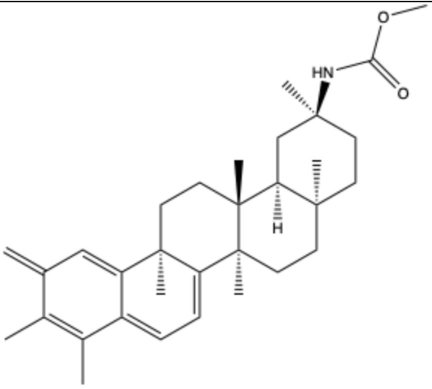	28.2 ± 2.8	-5.79
EA5	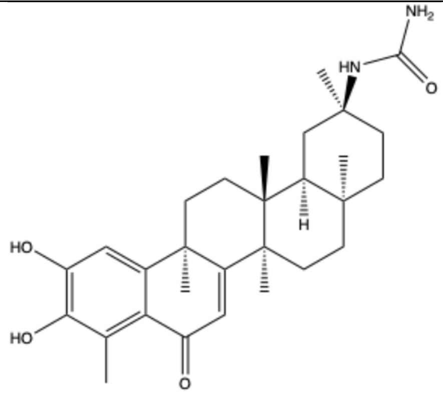	27.1 ± 3.0	-5.48
Rezvilutamide	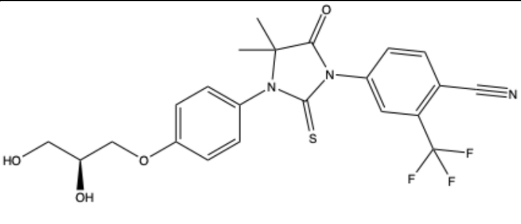	10.1 ± 1.9	-7.09

TAK-243	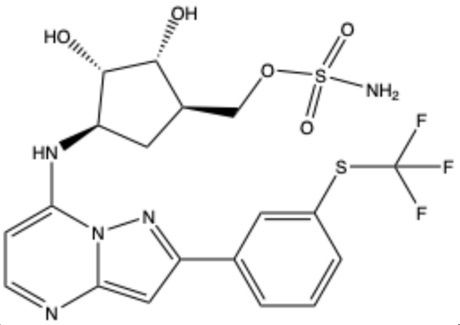	≤10	-6.50
Roflumilast	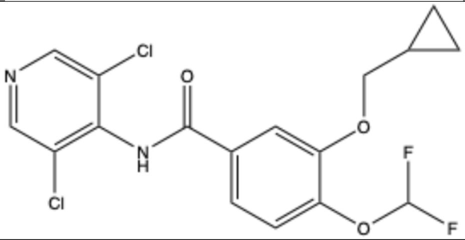	≤10	-5.71
Asunaprevir	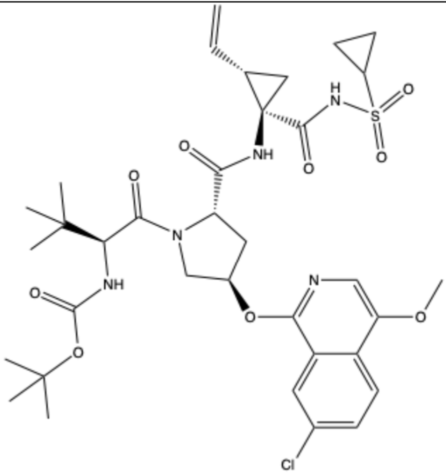	≤10	-5.60
Cariprazine	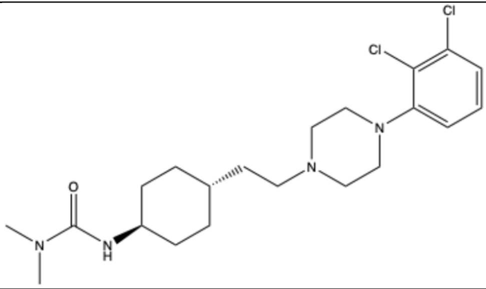	≤10	-6.25
EA9	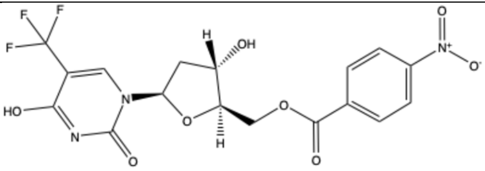	≤10	-7.59
Dovitinib	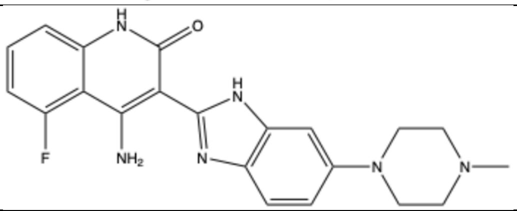	≤10	-5.85

EA19	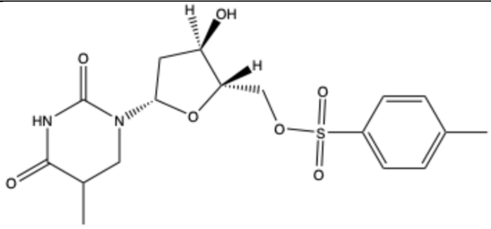	≤10	-7.52
EA11	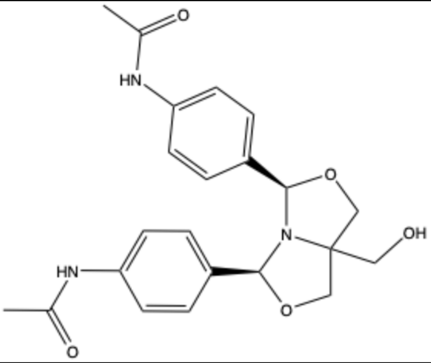	NA	-7.13
EA31	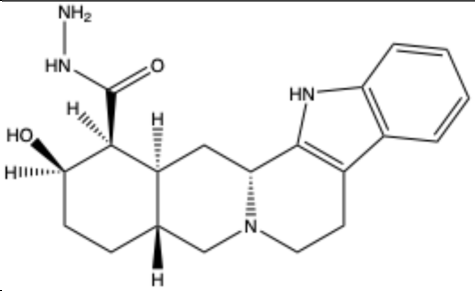	NA	-6.8
EA15	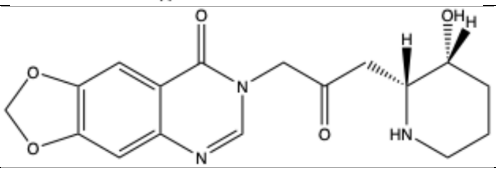	NA	-7.37

Table S5. Nonlinear regression parameters for dose–response analyses of the four validated compounds against USP7-FL and USP7-CD. NE: confidence limit not estimable by nonlinear regression because the profile likelihood did not converge within the tested parameter range.

Compound	USP7 construct	IC_{50} (μM)	95% CI (μM)	Hill slope	R^2
Montelukast sodium	USP7-FL	29.00	NE–30.08	−6.48	0.9929
	USP7-CD	15.71	13.53–17.37	−5.69	0.9890
Candesartan cilexetil	USP7-FL	32.89	30.01–33.54	−5.62	0.9897
	USP7-CD	20.35	18.87–NE	−5.36	0.9928
Closantel	USP7-FL	18.15	17.04–NE	−6.27	0.9951
	USP7-CD	10.08	9.75–10.66	−4.63	0.9884
Rafoxanide	USP7-FL	25.00	24.39–25.36	−21.98	0.9943
	USP7-CD	23.44	22.46–NE	−5.72	0.9902
